# Out-group animosity drives engagement on social media

**DOI:** 10.1073/pnas.2024292118

**Published:** 2021-06-23

**Authors:** Steve Rathje, Jay J. Van Bavel, Sander van der Linden

**Affiliations:** ^a^Department of Psychology, University of Cambridge, Cambridge CB2 3RQ, United Kingdom;; ^b^Department of Psychology, Center for Neural Science, New York University, New York, NY 10003

**Keywords:** social media, polarization, intergroup, out-group, social identity

## Abstract

There has been growing concern about the role social media plays in political polarization. We investigated whether out-group animosity was particularly successful at generating engagement on two of the largest social media platforms: Facebook and Twitter. Analyzing posts from news media accounts and US congressional members (*n* = 2,730,215), we found that posts about the political out-group were shared or retweeted about twice as often as posts about the in-group. Each individual term referring to the political out-group increased the odds of a social media post being shared by 67%. Out-group language consistently emerged as the strongest predictor of shares and retweets: the average effect size of out-group language was about 4.8 times as strong as that of negative affect language and about 6.7 times as strong as that of moral-emotional language—both established predictors of social media engagement. Language about the out-group was a very strong predictor of “angry” reactions (the most popular reactions across all datasets), and language about the in-group was a strong predictor of “love” reactions, reflecting in-group favoritism and out-group derogation. This out-group effect was not moderated by political orientation or social media platform, but stronger effects were found among political leaders than among news media accounts. In sum, out-group language is the strongest predictor of social media engagement across all relevant predictors measured, suggesting that social media may be creating perverse incentives for content expressing out-group animosity.

## Significance

Almost four billion people around the world now use social media platforms such as Facebook and Twitter, and social media is one of the primary ways people access news or receive communications from politicians. However, social media may be creating perverse incentives for divisive content because this content is particularly likely to go “viral.” We report evidence that posts about political opponents are substantially more likely to be shared on social media and that this out-group effect is much stronger than other established predictors of social media sharing, such as emotional language. These findings contribute to scholarly debates about the role of social media in political polarization and can inform solutions for creating healthier social media environments.

According to a recent article in the Wall Street Journal, a Facebook research team warned the company in 2018 that their “algorithms exploit the human brain’s attraction to divisiveness.” This research was allegedly shut down by Facebook executives, and Facebook declined to implement changes proposed by the research team to make the platform less divisive (1). This article is consistent with concerns that social media might be incentivizing the spread of polarizing content. For instance, Twitter CEO Jack Dorsey has expressed concern about the popularity of “dunking” (i.e., mocking or denigrating one’s enemies) on the platform (2). These concerns have become particularly relevant as social media rhetoric appears to have incited real-world violence, such as the recent storming of the US Capital (3). We sought to investigate whether out-group animosity was associated with increased virality on two of the largest social media platforms: Facebook and Twitter.

A growing body research has examined the potential role of social media in exacerbating political polarization (4, 5). A large portion of this work has centered on the position that social media sorts us into “echo chambers” or “filter bubbles” that selectively expose people to content that aligns with their preexisting beliefs (6–11). However, some recent scholarship questions whether the “echo chamber” narrative has been exaggerated (12, 13). Some experiments suggest that social media can indeed increase polarization. For example, temporarily deactivating Facebook can reduce polarization on policy issues (14). However, other work suggests that polarization has grown the most among older demographic groups, who are the least likely to use social media (15), albeit the most likely to vote. As such, there is an open debate about the role of social media in political polarization and intergroup conflict.

Other research has examined the features of social media posts that predict “virality” online. Much of the literature focuses on the role of emotion in social media sharing. High-arousal emotions, whether they are positive (e.g., awe) or negative (e.g., anger or outrage), contribute to the sharing of content online (16–20). Tweets expressing moral and emotional content are more likely to be retweeted within online political conversations, especially by members of one’s political in-group (21, 22). On Facebook, posts by politicians that express “indignant disagreement” receive more likes and shares (23), and negative news tends to spread farther on Twitter (24). Moreover, false rumors spread farther and faster on Twitter than true ones, especially in the domain of politics, possibly because they are more likely to express emotions such as surprise and fear (25).

Yet, to our knowledge, little research has investigated how social identity motives contribute to online virality. Group identities are hypersalient on social media, especially in the context of online political or moral discussions (26). For example, an analysis of Twitter accounts found that people are increasingly categorizing themselves by their political identities in their Twitter bios over time, providing a public signal of their social identity (27). Additionally, since sharing behavior is public, it can reflect self-conscious identity presentation (28, 29). According to social identity theory (30) and self-categorization theory (31), when group identities are highly salient, this can lead individuals to align themselves more with their fellow in-group members, facilitating in-group favoritism and out-group derogation in order to maintain a positive sense of group distinctiveness (32). Thus, messages that fulfill group-based identity motives may receive more engagement online. As an anecdotal example, executives at the website Buzzfeed, which specializes in creating viral content, reportedly noticed that identity-related content contributed to virality and began creating articles appealing to specific group identities (33).

People may process information in a manner that is consistent with their partisan identities, prior beliefs, and motivations, a process known as motivated cognition (34–37). Scholars noted early on that the degree to which individuals identify with their political party “raises a perceptual screen through which the individual tends to see what is favorable to his [or her] partisan orientation” (38). Partisan motivations have been hypothesized to influence online behavior, such as the sharing of true and false news online (39, 40). Accordingly, we suggest that just as people engage in motivated cognition—processing information in a way that supports their beliefs—people may also engage in motivated tweeting (or sharing, liking, or retweeting), selectively interacting with and attending to content that aligns with their partisan identity motivations. There is already evidence suggesting that people selectively follow (41) and retweet (10, 42) in-group members at much higher rates than out-group members.

In polarized political contexts, out-group animosity may be a more successful strategy for expressing one’s partisan identity and generating engaging content than in-group favoritism. Political polarization has been growing rapidly in the United States over the past few decades. Affective polarization, which reflects dislike of people in the opposing political party as compared to one’s own party, has most strikingly increased (43), and ideological polarization may have increased as well (though this is still a topic of debate) (44). This growth in affective polarization is driven primarily by increasing out-party animosity (rather than increasing in-party warmth)—a phenomenon known as “negative partisanship” (45). According to recently released American National Election Studies data, affective polarization grew particularly steeply from 2016 to 2020, reaching its highest point in 40 y. Out-party animosity, more so than in-party warmth, has also become a more powerful predictor of important behaviors, such as voting behavior (46) and the sharing of political fake news (39). When out-party animosity is strong, partisans are motivated to distinguish themselves from the out-party (by, for instance, holding opinions that are distinct from the out-party) (47). While some research suggests that out-group cues might be more powerful than in-group cues (48), there is still debate about the extent to which partisan belief and behavior is driven by in-group favoritism versus out-group derogation (49). A limitation of prior research is that much of it is based on self-report surveys, and so it remains unknown how expressions of in-group favoritism or out-group animosity play out in a social media context—or whether one might be a more powerful contributor to virality than the other.

We investigated the role that political in-group and out-group language, as well as emotional language, play in predicting online engagement in a large sample of posts from news media accounts and US congressional members (*n* = 2,730,215). We sought to examine this on both Facebook and Twitter since they are two of the world’s largest and most influential social media companies and constitute around three billion users out of nearly four billion total social media users worldwide (50). Specifically, we were interested in 1) how political in-group and out-group language compared to other established predictors of social media engagement, 2) whether in-group or out-group language was a better predictor of shares and retweets, and 3) whether out-group terms were associated with negative emotions (as measured by the six Facebook “reactions”), and whether in-group terms were associated with positive emotions, reflecting patterns of out-party derogation and in-group favoritism. Finally, 4) we wanted to see if these findings applied to both news sources and political leaders, who often have an outsized influence on social discourse as well as policy change.

## Results

To analyze these questions, we examined large datasets of tweets and Facebook posts from liberal media sources and conservative media sources (as defined by https://www.allsides.com/media-bias/media-bias-ratings, see *Materials and Methods* and *SI Appendix*, Fig. S3) as well as liberal (i.e., Democrat) and conservative (i.e., Republican) members of Congress. Specifically, we counted how many words in each tweet or Facebook post referred to 1) liberal, 2) conservative, or included 3) negative emotion, 4) positive emotion, or 5) moral-emotional language. To measure reference to a liberal or conservative, we use a list of the top 100 most famous Democrat and Republican politicians as defined by YouGov, a list of all the Democrat and Republican congressional members and a list of liberal and conservative identity terms (e.g., “left-wing,” “conservative,” or “far-right”), which have been used in prior research (27, 39). We also used previously validated dictionaries of negative affect, positive affect, and moral-emotional language (21, 51). Adapting prior methods used in similar studies (22), we fit regression models to examine how language about the out-group, language about the in-group, as well as language expressing various emotions (positive affect, negative affect, and moral-emotional language) predicted retweets and Facebook shares, controlling for various factors known to be correlated with retweet or sharing rate, such as whether a tweet is a retweet (for the Twitter datasets only), whether a message contained a URL or media, and how many followers or likes the account had. More details are in *Materials and Methods*. Data and code are available on the Open Science Framework (OSF) at https://osf.io/py9u4/.

### Study 1: Major Media Outlets.

In Study 1, we looked at liberal (e.g., New York Times, MSNBC) and conservative (e.g., Fox News, Breitbart) media accounts from Facebook (*n* = 599,999 posts) and Twitter (*n* = 227,229 posts). First, we looked at the effect of emotional language on message diffusion. Controlling for all other factors, each additional negative affect word was associated with a 5 to 8% increase in shares and retweets, except in the conservative media Facebook dataset, where it decreased shares by around 2% (exp(*b*) = 0.98, 95% CI = [0.98, 0.99], *P* < 0.001). Positive affect language was consistently associated with a decrease in shares and retweet rates by about 2 to 11% across datasets. This largely replicates prior work on the negativity bias in news headlines (24, 52). Additionally, moral-emotional words consistently increased shares and retweets in all datasets by 10 to 17%, replicating prior work on the moral contagion effect with similar effect sizes and extending on this work by showing that moral contagion operates on multiple social media platforms, including Facebook (21).

To test our primary questions, we looked at how political in-group language predicted diffusion. In the liberal news media accounts on Twitter, political in-group words were associated with increased retweet rate (exp(*b*) = 1.10, 95% CI = [1.09, 1.12]). On Facebook, however, there was no equivalent effect of political in-group language (exp(*b*) = 1.00, 95% CI = [0.99, 1.00]). In the conservative media Twitter accounts, political in-group (conservative) words increased retweet rate (exp(*b*) = 1.23, 95% CI = [1.20, 1.26], *P* < 0.001), and this effect was similar on Facebook (exp(*b*) = 1.37, 95% CI = [1.35, 1.38]), *P* < 0.001). In sum, political in-group words led to an estimated 0 to 37% increase in diffusion per word across all four media datasets.

We then looked at the effects of political out-group language. In the liberal media Twitter accounts, out-group language was a strong predictor of retweets (exp(*b*) = 1.46, 95% CI = [1.44, 1.48]). This effect was similar on Facebook, with out-group language leading to increased shares (exp(*b*) = 1.57, 95% CI = [1.55, 1.58]). In the conservative media Twitter accounts, out-group language increased retweet rate (exp(*b*) = 1.29, 95% CI = [1.26, 1.31], *P* < 0.001), and this effect was similar on Facebook (exp(*b*) = 1.35, 95% CI = [1.34, 1.36], *P* < 0.001). Thus, across datasets, out-group language led to a 35 to 57% increase in diffusion per additional out-group word.

**Fig. 1.**
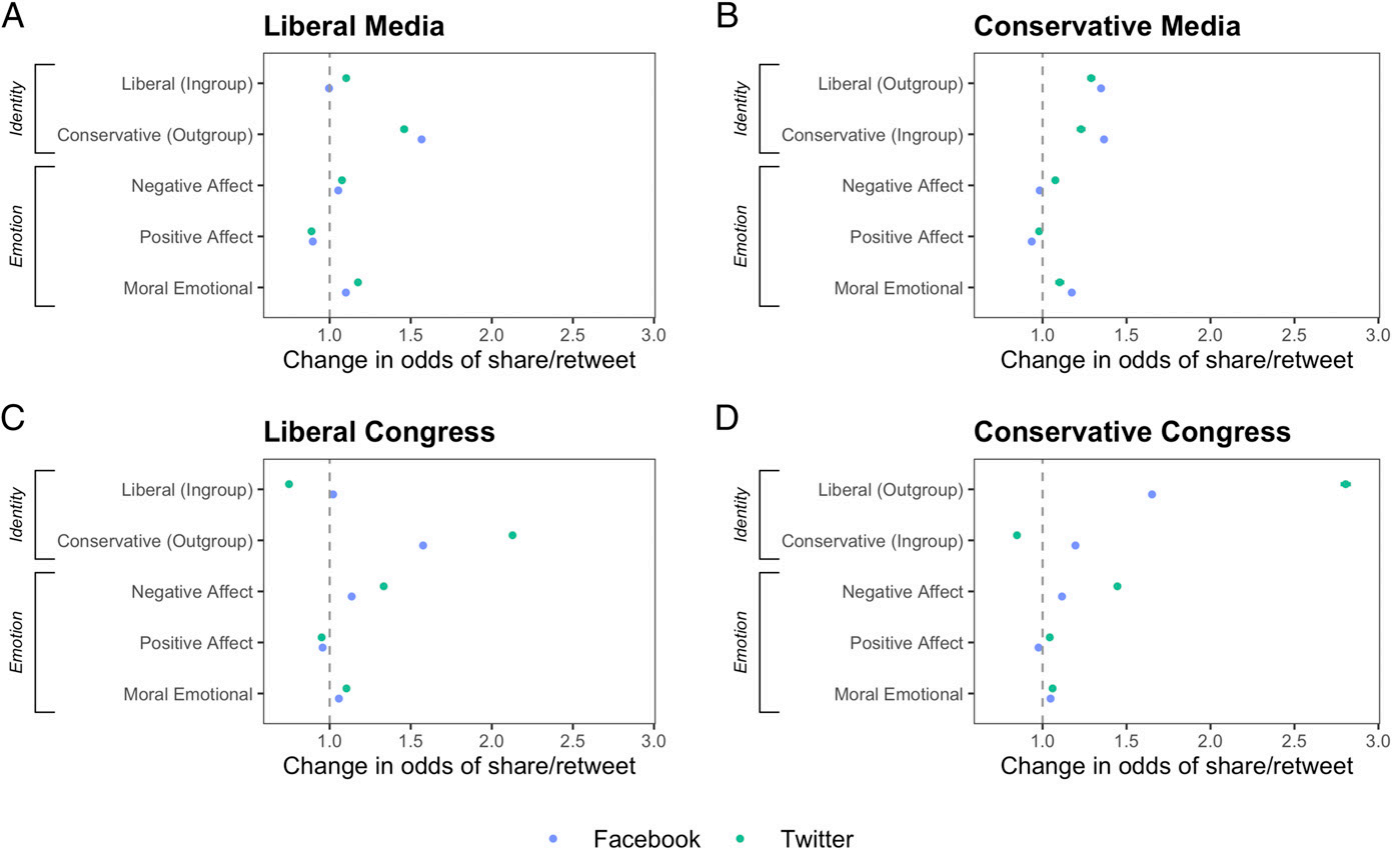
Political out-group words were the strongest predictors of shares and retweets from both liberal and conservative news media accounts (*A* and *B*, respectively) and liberal and conservative congress member accounts (*C* and *D*, respectively) on Facebook and Twitter. By comparison, political in-group words, as well as measures of discrete emotions, such as positive emotion words, negative emotion words, and moral-emotional words, were relatively weak predictors of shares and retweets. Error bars represent 95% CI (though error bars are small).

Descriptively, the effect sizes of political out-group language are generally larger than those of in-group language and considerably larger than those of any of the emotional dictionaries. The full regression models are reported in *SI Appendix*, Table S1 and are plotted visually in Fig. 1. The results were similar when the control variables were removed (*SI Appendix*, Table S3) and when the models were rerun with cluster-robust SEs with each media account representing a different cluster (*SI Appendix*, Table S4). To further probe the importance of each predictor in the model, we calculated a relative importance analysis (*SI Appendix*, Table S4). In each of the models, out-group words had the highest “lmg” values (an estimate of the *R*^*2*^ contributed by each predictor) of all five of the key predictors. Thus, political out-group language appears to be the most powerful predictor of engagement of all factors measured.

**Fig. 2.**
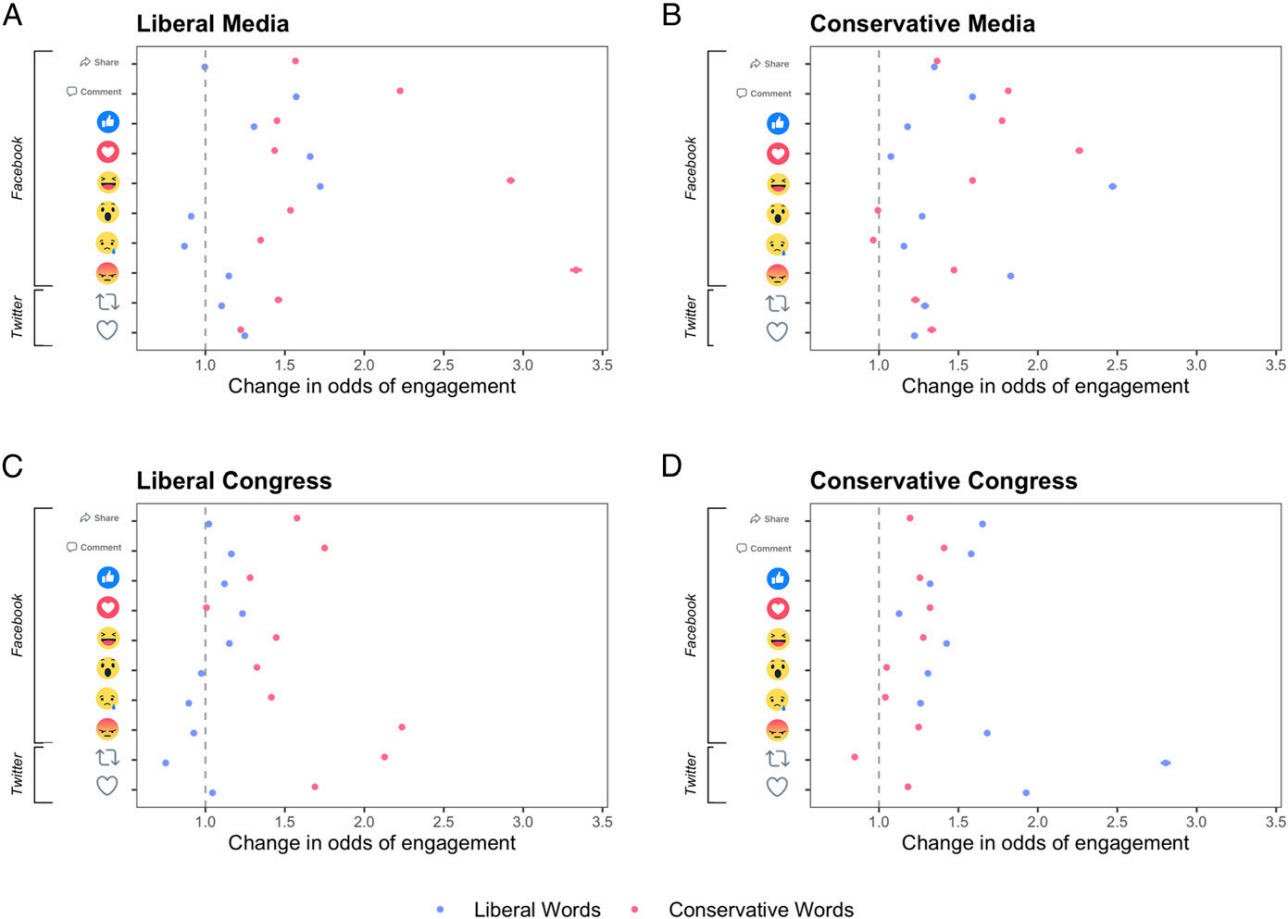
In-group and out-group words predicted different types of engagement in both the liberal and conservative news media accounts (*A* and *B*, respectively) and congress accounts (*C* and *D*, respectively). Political out-group words were strong predictors of shares, comments, “haha,” and “angry” reactions, whereas in-group words were strong predictors of “love” reactions. Reactions are shown as they are shown on the Facebook and Twitter platforms (from *Top* to *Bottom*: share, comment, like, heart, haha, wow, sad, angry, retweet, and favorite). Error bars represent 95% CI (though error bars are small).

We next assessed the valence generated by posts with political out-group language. We expected posts about the out-group to evoke negative emotions such as anger or outrage and posts about the in-group to evoke positive emotions. Examples of some of the most popular tweets and Facebook posts containing out-group terms are in Table 1. Descriptively, these posts appeared to be very negative. To assess the valence of these posts more systematically, we examined how out-group language predicted each of the six “reactions” (like, love, haha, sad, wow, and angry) available on Facebook. We assumed that the “angry” reaction was a reasonable proxy for feelings of out-group animosity, outrage, and anger, and the “love” reaction was a reasonable proxy for feelings of in-group love. These results are plotted in Fig. 2, and full regression models are shown in *SI Appendix*, Tables S6 and S7.

**Table 1.** Example tweets and Facebook posts

Dataset	Liberal	Shares/retweets	Conservative	Shares/retweets
Media (Facebook)	BREAKING: PRESIDENT **TRUMP** HAS BEEN IMPEACHED.	82,886	Reported **Antifa** Protester tries a sucker punch and it doesn't go so well…	71,482
Media (Twitter)	Vice President **Mike Pence** blatantly lied to reporters about the trajectory of COVID-19 cases in Oklahoma, where President **Trump** is scheduled to hold a large campaign rally on Saturday.	8,793	Every American needs to see Joe **Biden**'s latest brain freeze.	15,354
Congress (Facebook)	Donald **Trump** has lied more than 3,000 times since taking office but **Republicans** refuse to say **Trump** is a liar. What's going on?	29,737	**Democrats** just passed a bill that would make it harder for American innovators to develop a COVID-19 vaccine.‬ Here’s what you need to know:‬	10,354
Congress (Twitter)	**Republicans** are saying they are being barred from the “secret” hearings. But here’s a list of every **Republican** who is allowed into the hearings.	41,541	RT to tell **Chuck Schumer** and **Nancy Pelosi** to STOP blocking critical funding for small businesses. The Paycheck Protection Program is about to run out of money—millions of jobs are hanging in the balance. Congress MUST ACT!	37,872

Examples of some of the most popular posts from each dataset, along with their shares and retweets at the time of data collection. Political out-group language is bolded.

As expected, political out-group language was a very strong predictor of “angry” reactions for both liberals (exp(*b*) = 3.33, 95% CI = [3.30, 3.37], *P* < 0.001) and for conservatives (exp(*b*) = 1.83, 95% CI = [1.81, 1.85], *P* < 0.001). Out-group words were also strong predictors of “haha” reactions for both groups (exp(b)_liberals_ = 2.92, 95% CI = [2.90, 2.95], *P* < 0.001; exp(*b*)_conservatives_ = 2.47, 95% CI = [2.45, 2.50], *P* < 0.001). Thus, posts about the out-group may generate engagement by inspiring negative emotions such as anger, outrage, or mockery. Strikingly, descriptive statistics (*SI Appendix*, Table S9) show that, on average, the angry reaction was the most popular of the six reactions for both liberals and conservatives in the news media accounts, consistent with the perspective that outrage is popular on online social networks (18). On the flip side, in-group words, as expected, strongly predicted love reactions for both liberals (exp(*b*) = 1.66, 95% CI = [1.64, 1.68], *P* < 0.001) and conservatives (exp(*b*) = 2.26, 95% CI = [2.24, 2.26], *P* < 0.001).

### Study 2: Congress Members.

In Study 2, we replicated the above results in a different context: tweets (*n* = 1,078,562) and Facebook posts (*n* = 825,424) by Democratic and Republican Congressional members. Given growing levels of polarization in Congress (53), and because political elites are often agenda setters who frame political debates and influence public opinion (54, 55), we thought this was an important additional context to investigate the virality of social media posts.

First, we looked at the effect of emotional language on virality. Negative affect language consistently increased retweet rate and shares across all datasets by 12 to 45% per negative affect word, with the effect size being largest in the conservative Twitter dataset (exp(*b*) = 1.45, 95% CI = [1.44, 1.45], *P* < 0.001). Similarly, moral-emotional language had a consistent positive effect across all datasets, increasing retweets and shares by roughly 5 to 10%. Positive affect language slightly decreased shares by roughly 2 to 5%, except in the conservative Twitter accounts (exp(*b*) = 1.04, 95% CI = [1.04, 1.05], *P* < 0.001). Replicating the results from Study 1, negative language and moral-emotional language were once again positively associated with diffusion, whereas positive affect language was negatively associated with it.

Next, we again looked at the effects of political in-group language. In the liberal congressional accounts, political in-group language decreased retweet rate on Twitter (exp(*b*) = 0.75, 95% CI = [0.75, 0.75], *P* < 0.001) and only slightly increased shares on Facebook (exp(*b*) = 1.02, 95% CI = [1.01, 1.03], *P* < 0.001). Similarly, in the conservative dataset, political in-group language decreased retweet rate on Twitter (exp(*b*) = 0.85, 95% = [0.84, 0.85], *P* < 0.001) and slightly increased shares on Facebook (exp(*b*) = 1.20, 95% = [1.19, 1.20], *P* < 0.001). In sum, political in-group language led to a mixed pattern of results across all four congressional datasets.

Replicating our findings with media accounts, political out-group language was a very large predictor of retweets in the liberal congressional Twitter accounts (exp(*b*) = 2.13), 95% CI = [2.11, 2.15], *P* < 0.001) and of shares in the liberal congressional Facebook accounts (exp(*b*) = 1.58, 95% CI = [1.57, 1.59], *P* < 0.001). The same was true in the conservative congressional Twitter accounts (exp(*b*) = 2.80, 95% CI = [2.77, 2.84], *P* < 0.001) and Facebook accounts (exp(*b*) = 1.65, 95% CI = [1.64, 1.67], *P* < 0.001). This effect translates into an estimated 65 to 180% increase in the odds of being shared per out-group word across datasets. Descriptively, these effect sizes are very large and larger than those found in the news media accounts. This might be due to the fact that members of Congress are explicitly identified with a political party and have a large partisan following.

To further explore the importance of political out-group language, we conducted another relative importance analysis (*SI Appendix*, Table S13). In each model, out-group language had the highest estimated *R*^*2*^ (“lmg”) value compared to the other key predictors (political in-group, negative, positive, and moral-emotional language). In other words, it was once again the most important predictor in each model.

When examining different types of engagement (e.g., the six Facebook reactions, reference *SI Appendix*, Tables S17 and S18 for more detail), we once again saw similar patterns to media outlets. Posts about the out-group strongly predicted negative reactions, such as “angry” reactions, for both liberals (exp(*b*) = 2.24, 95% CI = [2.22, 2.25], *P* < 0.001) and for conservatives (exp(*b*) = 1.68, 95% CI = [1.67, 1.69], *P* < 0.001). On the other hand, posts about the political in-group predicted “love” reactions for both liberals (exp(*b*) = 1.23, 95% CI = [1.22, 1.24], *P* < 0.001) and for conservatives (exp(*b*) = 1.32, 95% CI = [1.31, 1.33], *P* < 0.001). Descriptive statistics (*SI Appendix*, Tables S19 and S20) again found that the angry reaction was generally the most popular reaction, although the “love” reaction slightly surpassed the angry reaction in popularity in the conservative dataset.

### Internal Meta-Analysis.

**Fig. 3.**
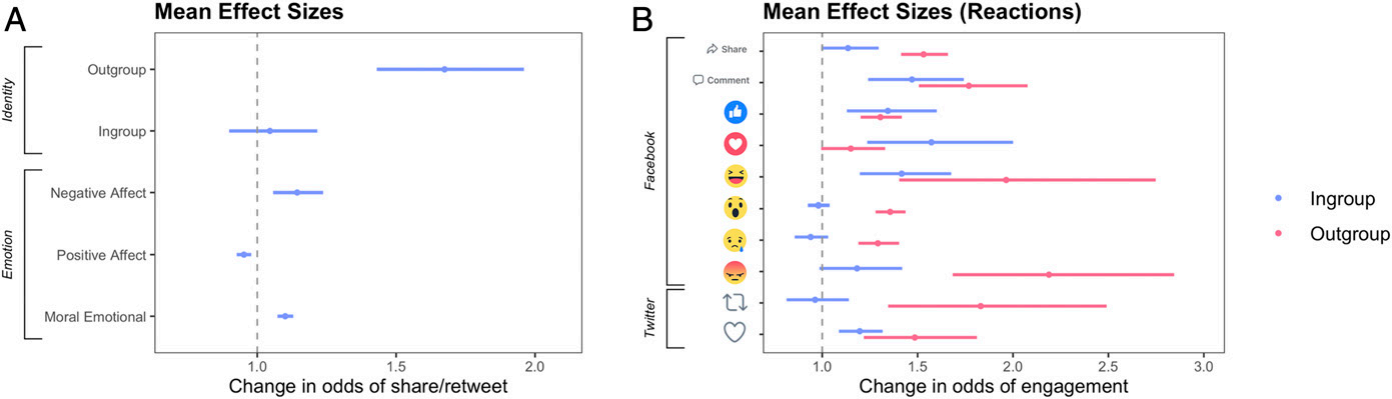
In *A*, the meta-analyzed effect sizes across all eight datasets are shown. The effect size of political out-group language was 4.7 times as large as that of negative affect language and 6.7 times as large as moral-emotional language. In *B*, the meta-analyzed effect sizes of in-group and out-group language on each of the Facebook reactions are shown. In-group language predicted “love” reactions, whereas out-group language strongly predicted “angry” reactions.

To estimate the average effect sizes across all eight datasets, we conducted a series of internal meta-analyses (Fig. 3*A* and *SI Appendix*, Table S22). We computed a random-effects meta-analysis (because we expected this effect to vary across contexts) and used the Dersimonian–Laird estimator. Across datasets, each political out-group word increased the odds of a retweet or share by about 67% (estimated exp(*b*) = 1.67, 95% CI = [1.43, 1.69], *P* < 0.001).* Political in-group language, on the other hand, did not have a statistically significant effect on shares and retweets (exp(*b*) = 1.05, 95% CI = [0.90, 1.22], *P* = 0.563). Negative affect language increased diffusion by about 14% per word (exp(*b*) = 1.14, 95% CI = [1.05, 1.24]), moral-emotional language increased diffusion by 10% per word (exp(*b*) = 1.10, 95% CI = [1.07, 1.13], *P* < 0.001), and positive affect language decreased diffusion by about 5% per word (exp(*b*) = 0.95, 95% CI = [0.93, 0.98], *P* < 0.001).

To put these effect sizes in context, the average percent increase in shares of political out-group language was about 4.8 times as large as that of negative affect language and about 6.7 times as large as that of moral-emotional language. While one might expect words that have clear political content (e.g., names of specific politicians) to be more predictive of social media shares than words that refer to general emotions (e.g., adjectives such as “bad”), this large effect size is notable, because negative emotion and moral-emotional language are well-established predictors of diffusion on social networks (19, 21, 22, 52) and have been the main focus of prior work looking at social media diffusion. Here, we show that the use of out-group terms (but not in-group terms) is a much stronger predictor of diffusion than various measurements of moral or emotional language.

We then analyzed a set of moderator variables in the meta-analysis. The effect of political out-group language on diffusion was not moderated by political orientation (exp(*b*) = 0.98, 95% CI = [0.75, 1.30], *P* = 0.187) nor by social media platform (exp(b) = 1.20, 95% CI = [0.91, 1.58], *P* = 0.910). However, it was moderated by whether the tweets came from members of Congress as opposed to news media accounts (exp(b) = 1.39, 95% CI = [0.91, 1.58], *P* < 0.018).^†^ Thus, we did not detect any ideological asymmetries in the internal meta-analysis nor did we find any key differences between social media platforms. However, the effect was clearly stronger among politicians than in the news media accounts, possibly because of the more explicitly partisan rhetoric of political leaders. The estimated effect size for the media datasets only was exp(*b*) = 1.41, 95% CI = [1.30, 1.54], *P* < 0.001 (a 41% increase) and the estimated effect size for the Congress datasets was exp(b) = 1.99, 95% CI = [1.53, 2.58], *P* < 0.001 (a 99% increase).

Because this analysis focused on the effect of each additional out-group word, we also conducted additional analyses where we examined how much a post with at least one out-group term diffused compared to a post with at least one in-group term, controlling for all the same relevant variables. Posts with both in-group and out-group terms, as well as posts with no in-group and out-group terms, were excluded from this analysis. Thus, we could directly compare how much posts about only the out-group (coded as 1) diffused compared to posts about only the in-group (coded as 0), following the methods of past research that have looked at how the diffusion of false news compares to true news (25). When meta-analyzed across all eight datasets, posts with at least one out-group word were more than twice as likely to be shared than posts with at least one in-group word (estimated exp(*b*) = 2.32, 95% CI = [1.57, 2.47], *P* < 0.001) (a 132% increase).

We also conducted internal meta-analyses using the same methods to report average effect sizes for each of the Facebook reactions (Fig. 3*B*). While all reactions are shown in Fig. 3, we focused specifically on “anger” and “love” reactions, as these most clearly indicate out-group animosity or in-group love. Out-group language was a very large predictor of the “angry” reaction across datasets (exp(*b*) = 2.19, 95% CI = [1.68, 2.84], *P* < 0.001), but in-group language was only marginally associated with the “angry” reaction (exp(*b*) = 1.18, 95% CI = [0.99, 0.42], *P* = 0.07). Furthermore, in-group language was strongly associated with the “love” reaction across datasets (exp(*b*) = 1.57, 95% CI = [1.24, 2.00], *P* < 0.001), whereas out-group language was not associated with the “love” reaction (exp(b) = 1.15, 95% CI = [1.00, 1.33], *P* = 0.059). Thus, out-group language appears to reflect out-group derogation, whereas in-group language reflects in-group favoritism. Furthermore, the effect size of out-group language predicting “angry” reactions was more than twice as big as the effect size of in-group language predicting “love” reactions, once again showing an out-group bias.

We wanted to test whether the effect of political out-group language was not driven by any specific words in particular (e.g., “Trump”). To examine this, we repeated our analysis with each of the three subdictionaries that made up the political out-group language dictionary: 1) the dictionaries of the Democratic and Republican “identity” terms (i.e., “Democrat,” “right-wing,” or “leftist”), 2) lists of the top 100 Democratic and Republican politicians as ranked by YouGov, along with their Twitter handles (or Facebook page names on Facebook), and 3) lists of all liberal and conservative congressional members, along with their Twitter handles (or Facebook page name on Facebook). We then meta-analyzed these results across all eight datasets.

Looking only at the “identity” terms, political out-group language led to an estimated 91% increase in the odds of being shared per word (exp(*b*) = 1.91, 95% CI = [1.38, 2.64], *P* < 0.001). An additional word from the top 100 most famous politicians dictionary led to an estimated 82% increase in the odds of being shared (exp(*b*) = 1.82, 95% CI = [1.53, 2.17], *P* < 0.001). Lastly, each additional word from the list of out-group congressional members led to a 43% increase in shares (exp(*b*) = 1.43, 95% CI = [1.23, 1.66], *P* < 0.001). In other words, whether referring to a general identity term, a famous politician, or a member of Congress, out-group language is a very strong predictor of diffusion. This helps validate that this phenomenon is not dependent on any one specific dictionary and is robust across specifications. The slightly smaller effect size of out-group congressional words may be due to the fact that many congressional members are not as widely known as the most famous politicians. Because of this, including the full list of congressional members in the main analysis may have led to a conservative estimation of the true effect size.

## Discussion

Across 2,730,215 total observations from Facebook and Twitter, we find that posts about the political out-group are consistently more likely to be shared than those about the political in-group. The effect of out-group language was the most important predictor of sharing behavior in posts from both news media accounts and politicians—considerably stronger than the effects of political in-group language or various discrete emotions, which have previously been the main focus when assessing what makes content go “viral” online (17, 26, 56). To contextualize this large effect, the percent increase in estimated shares associated with out-group language was 4.8 times as big as that of negative affect language and 6.7 times as big as that of moral-emotional language—previously established predictors of message diffusion online.

This out-group effect was also robust against different ways of operationalizing the out-group, suggesting that this pattern of results is not primarily driven by the mention of specific terms or particularly divisive politicians, such as Donald Trump. The effect was not moderated by political orientation or by social media platform when we measured findings in an internal meta-analysis. However, the effect of out-group language was considerably stronger among politicians than in the news media accounts, perhaps because of the more explicitly partisan rhetoric among political elites (57, 58) and their followers. Additionally, given prior concerns that much of social media research focuses predominantly on Twitter due to the relatively easy accessibility of Twitter data (4), it is notable that similar patterns were found on both Twitter and Facebook.

Political in-group and out-group language also generated distinctly different forms of engagement, reflecting clear patterns of in-group favoritism and out-group derogation. For instance, out-group language strongly predicted “angry” reactions (as well as “haha” reactions, comments, and shares), and in-group language strongly predicted love reactions. Though, notably, out-group language was about twice as strong a predictor of “angry” reactions as in-group words were of “love” reactions. Thus, posts about the out-group may be so successful because they appeal to emotions such as anger, outrage, and mockery. Indeed, the “angry” reaction was the most popular reaction on Facebook in seven of the eight datasets analyzed.

This research is consistent with prior research showing that expressions of moral outrage (which involve emotions such as anger or disgust) are particularly likely to go viral (18, 26), but it expands on that work by illuminating the role of out-group animosity in eliciting outrage. The current research reveals the key role that out-group identity language played in predicting sharing behavior above and beyond emotional words alone. In fact, in *SI Appendix*, Tables S6, S7, S17, and S18, we found that emotional language was weakly associated with the various Facebook reactions, suggesting that out-of-context emotional language used in a post may not be the most precise way to measure actual emotions evoked by a social media post.

These results demonstrate how the predictions of social identity theory play out in a modern social media context (see ref. 24). As expected, posts that appeal to identity-based motives tend to receive more engagement in online social networks. Additionally, there is also a strong asymmetry such that out-group negativity is stronger than in-group positivity, reflecting the current state of negative partisanship in the United States (45, 46). These results also expand on prior work on the motives behind social media sharing. Social media sharing often reflects a desire to maintain a positive self-presentation (17, 29). This can lead to different outcomes depending on the context, social norms, and design features of one’s online network (59), since strategies to maintain a positive self-image may differ by context (26, 60). While many studies find a negativity bias in online sharing (19, 52), there are some contexts where positive content is shared more often. For instance, the New York Times most emailed list tends to have more positive content (29, 56), as do viral articles about science (56). However, in online contexts where political identity is highly salient, and where political conflict is driven by negative partisanship, the best way to maintain an image of a good in-group member and to distinguish oneself from the out-group may be to share expressions of out-party animosity (47). Additionally, other work has found that words that predict virality (such as moral or emotional words) are prioritized in early visual attention (61). Political identity-relevant content may also be similarly attention grabbing, especially when political conflicts have become excessively negative and moralized (46).

While much of the literature on social media and political polarization has focused on the formation of echo chambers, the finding that social media amplifies out-group animosity might be more concerning than the formation of echo chambers alone. Even if people are exposed to more cross-partisan content than expected (6, 12), our findings suggest that opposing views on social media may be excessively negative about one’s own side. This may help explain why exposure to opposing views on Twitter can actually increase political polarization (62). Thus, the severity of online echo chambers appears to be a less important issue than the kind of content that tends to surface at the top of one’s feed, since exposure to divisive in-party or out-party voices is unlikely to be productive. While future experimental work is needed to examine the consequences of these trends, the amplification of divisive posts on social media—from both in-party and out-party sources—may be playing a role in rising political polarization.

This big data approach comes with many benefits, such as allowing us to understand how political identity contributes to engagement with online content and thus has high ecological validity. However, this approach also comes with several limitations. While these results may be consistent with theoretical predictions, they are correlational, and further experimental work should be conducted to determine causation and help clarify why content about out-group identities is engaging in online political conversations. Additionally, while we found this effect among contexts on two of the largest social media platforms, we were unable to follow up certain important questions, such as who is producing this engagement, due to data access limitations.

It is important to note that the data we observed are likely reflective of a specific time frame, namely the years leading up to the 2020 election. Since the language of political elites can change depending on which party is in power (63), and the United States is at historically high levels of polarization (44), it is unclear whether these results would generalize to different time periods or nations. It is also unclear how algorithmic choices on the part of Facebook or Twitter might contribute to the amplification of out-group animosity, since social media companies are not transparent about how their algorithms work. Despite these drawbacks, this study reveals a consequential trend playing out within two of the most influential social networks, inspiring many questions for future research.

This research is also important on a practical level. Social media is encroaching on more aspects of our lives, becoming one of the main ways in which people consume news and interact with politicians (64). Since the social media ecosystem operates as an attention economy (65) whereby users, politicians, and brands fight for attention and engagement, understanding what drives virality is crucial. Virality can contribute to the success of a social movement, business, or political campaign (20), so people have strong incentives to generate viral, engaging content. Virality is also essential for social media companies, as the business model of social media is grounded in generating engagement with the platform, which leads to advertising revenue. When the chief goal is virality, this may create negative externalities in the form of polarizing, hyperpartisan, or false content. Indeed, false political news tends to be highly negative about the outgroup (39), so our findings may be relevant to the viral spread of misinformation online. Content expressing out-group animosity may be good at generating superficial engagement while ultimately harming individuals, political parties, or society in the long-term.

The design structure of social media platforms may be creating perverse incentives for polarizing content when users do not truly want this. For instance, people report that they do not want political leaders to express partisan animus (66), but our results suggest this content receives the most engagement. As further illustration of these perverse incentives, the New York Times reported on internal research from Facebook finding that posts that users rated as “bad for the world” received more engagement. When Facebook tested a feature to down-rank posts that were rated as “bad for the world,” engagement decreased, and Facebook ultimately chose not to approve the feature (67). Thus, social media companies may be reluctant to implement features that could reduce polarization due to their strong financial incentives to maintain user engagement.

## Conclusion

Understanding the factors that make social media posts go “viral” online can help to create better social media environments. While social media platforms are not fully transparent about how their algorithmic ranking system works, Facebook announced in a post titled “Bringing People Closer Together” that it was changing its algorithm ranking system to value “deeper” forms of engagement, such as reactions and comments (68). Ironically, posts about the political out-group were particularly effective at generating comments and reactions (particularly the “angry” reaction, the most popular reaction across our studies). In other words, these algorithmic changes made under the guise of bringing people closer together may have helped prioritize posts including out-group animosity. In addition to informing algorithmic changes (69), this research might inform other design changes (70), or policy changes that can be implemented to improve social media conversations, as well as future research on the role of social identity in online engagement. Amid widespread discussion that social media may be contributing to discord and polarization, our work reveals how out-group animosity predicts virality in two of the largest social networks.

## Materials and Methods

All methods were approved by the University of Cambridge Research Ethics Committee. For Study 1, we collected tweets from several news media accounts across the political spectrum using the R package “rtweet” and the Twitter API (application programming interface). After collecting up to 3,200 of the most recent tweets from each account (the total amount permitted by the Twitter API), we were left with a total of 227,229 total tweets for analysis. These news media accounts were chosen because they were classified by the All Sides Media Bias Chart (*SI Appendix*, Fig. S3), which aims to identify the political bias of various news sources. This allowed us to split the dataset into tweets from liberal (*n* = 143,702) and conservative (*n* = 83,527) media sources. In Study 2, we analyzed tweets from all members of Congress (up to 3,200 tweets per member). We split the dataset into tweets from Democratic (*n* = 747,675) and Republican (*n* = 611,292) US congressional members.

Facebook data were retrieved through a partnership with Crowdtangle, a tool owned by Facebook that aggregates data from public pages, and Social Science One, an organization that forms partnerships with industry and social science researchers. Using the Crowdtangle platform, we created lists of the same liberal and conservative media accounts from https://www.allsides.com/media-bias/media-bias-ratings and downloaded the 300,000 most recent posts from these lists of media accounts. We also used official lists assembled by the Crowdtangle staff of the current Democrat and Republican US House of Representative and Senate Members. After combining the downloaded lists of the US House of Representatives and US Senate, we were left with 366,842 liberal Congress Facebook posts and 458,582 conservative Congress Facebook posts. All data were retrieved during 2020, and the majority of the observations for the media accounts range from 2018 to 2020 and range from 2016 to 2020 in the congressional accounts. More information about the exact timeline that the tweets and Facebook posts reflect is in *SI Appendix*, Fig. S2. Data and code are available on the OSF at https://osf.io/py9u4/, though text of individual social media posts could not be shared for privacy reasons. We determined our sample size and exclusions in advance of analyzing the data and, where possible, kept the analysis methods as close as possible for Twitter and Facebook.

We used the R package “quanteda” to analyze Twitter and Facebook text. During text preprocessing, we removed punctuation, URLs, and numbers. To classify whether a specific post was referring to a liberal or a conservative, we adapted previously used dictionaries that referred to words associated with liberals or conservatives (39). Specifically, these dictionaries included 1) a list of the top 100 most famous Democratic and Republican politicians according to YouGov, along with their Twitter handles (or Facebook page names for the Facebook datasets) (e.g., “Trump,” “Pete Buttigieg,” “@realDonaldTrump”); 2) a list of the current Democratic and Republican (but not independent) US Congressional members (532 total) along with their Twitter and Facebook names (e.g., “Amy Klobuchar,” “Tom Cotton”); and 3) a list of about 10 terms associated with Democratic (e.g., “liberal,” “democrat,” or “leftist”) or Republican identity (e.g., “conservative,” “republican,” or “ring-wing”). We then assigned each tweet a count for words that matched our Republican and Democrat dictionaries (for instance, if a tweet mentioned two words in the “Republican” dictionary, it would receive a score of “2” in that category). We also used previously validated dictionaries that counted the number of positive and negative affect words per post (51) and the number of moral-emotional words per post (22). All dictionaries are available on the OSF (https://osf.io/py9u4/), except for the positive and negative affect dictionaries, which are proprietary and must be purchased through the program LIWC (Linguistic Inquiry and Word Count) (51).

In each dataset, adapting prior methods (22), we fit ordinary least squares regression models to examine how language about the out-group, language about the in-group, as well as language expressing various emotions (positive affect, negative affect, and moral-emotional language) predicted retweet rates. We controlled for whether a post contained a URL, media (i.e., photo or video), the number of followers each account had, and whether a tweet was a retweet. All variables were mean-centered using the R package “jtools.” Following prior work (22), we log-transformed the retweet-count and share outcome variables to account for the fact that these variables are typically skewed. We applied the same models to each of the individual Facebook reactions to assess different forms of engagement. Afterward, we conducted several random-effects internal meta-analyses using the R package “meta.” Analyses were performed using R version 4.0.1.

## Supplementary Material

Supplementary File

## Data Availability

Anonymized partial social media data and analysis code are available on OSF (https://osf.io/py9u4/). Some data could not be included (e.g., the raw social media text and usernames) due to privacy concerns.

## Footnotes

*Using different estimators for the meta-analysis did not yield different results. For instance, using the Empirical Bayes estimator led to the following estimated effect size: exp(b) = 1.67, 95% CI = [1.40, 2.00], *P* < 0.001, which is the same effect size with a slightly larger CI.

^†^For the moderation analysis, liberal was coded as 1, conservative was coded as 0, Twitter was coded as 1, Facebook was coded as 0, Congress was coded as 1, and media was coded as 0.
