# Supplementary material for: Out-group animosity drives engagement on social media
Source: Proc Natl Acad Sci U S A. 2021 Jun 23;118(26):e2024292118. doi: 10.1073/pnas.2024292118 (PMC8256037; doi:10.1073/pnas.2024292118)
Supplement: Supplementary File [file pnas.2024292118.sapp.pdf]

PNAS
www.pnas.org

Supplementary Information for
Outgroup animosity drives engagement on social media

Steve Rathje[1], Jay J. Van Bavel[2], Sander van der Linden[1]
[1]University of Cambridge, [2]New York University
*Steve Rathje and Sander van der Linden

Corresponding Author Emails:
sjr210@cam.ac.uk and sander.vanderlinden@psychol.cam.ac.uk

This file includes:

Table S1. Full Regression Models for Study 1.
Table S2. VIFS for Study 1.
Table S3. Study 1 Regression Models Without Control Variables
Table S4. VIFS for Study 1 Regression Without Control Variables
Table S5. Study 1 Robustness Check (Cluster Robust Standard Errors)
Table S6. Liberal Media Reactions Regression Analysis
Table S7. Conservative Media Reactions Regression Analysis
Table S8. Study 1 Descriptive Statistics
Table S9. Liberal Media Facebook Reactions
Table S10. Conservative Media Correlations and Descriptive Statistics
Table S11. Study 2 Regression Models
Table S12. VIFS to Study 2
Table S13. Study 2 Models Without Control Variables
Table S14. VIFS for Study 2 Models Without Control Variables
Table S15. Study 2 With Cluster Robust Standard Errors
Table S16. Study 2 Relative Importance Analysis
Table S17. Study 2 Conservative Congress Facebook Reactions
Table S18. Study 2 Liberal Congress Facebook Reactions
Table S19. Liberal Congress Facebook Reactions
Table S20. Conservative Congress Facebook Reactions
Table S21. Descriptive Statistics – Congress
Table S22. Meta-Analyzed Effect Sizes (Facebook Reactions)
Figure S1. Pages Associated with the Most Engagement on Facebook and Twitter.
Figure S2. Histograms of the time the tweets and Facebook posts were created.
Figure S3: AllSides Media Bias Chart.

Table S1. Full Regression Models for Study 1.

	Twitter		Facebook	
	Liberal	Conservative	Liberal	Conservative
(Intercept)	38.04 ***	68.68 ***	41.96 ***	19.75 ***
	[36.71, 39.42]	[66.25, 71.20]	[30.85, 57.08]	[17.74, 22.00]
Democrat	1.10 ***	1.29 ***	1.00	1.35 ***
	[1.09, 1.12]	[1.26, 1.31]	[0.99, 1.00]	[1.34, 1.36]
Republican	1.46 ***	1.23 ***	1.57 ***	1.37 ***
	[1.44, 1.48]	[1.20, 1.26]	[1.55, 1.58]	[1.35, 1.38]
NegativeAffect	1.08 ***	1.08 ***	1.05 ***	0.98 ***
	[1.07, 1.09]	[1.06, 1.09]	[1.05, 1.06]	[0.98, 0.99]
PositiveAffect	0.89 ***	0.98 **	0.90 ***	0.94 ***
	[0.88, 0.90]	[0.97, 0.99]	[0.89, 0.90]	[0.93, 0.94]
MoralEmotional	1.17 ***	1.10 ***	1.10 ***	1.17 ***
	[1.16, 1.19]	[1.07, 1.13]	[1.09, 1.11]	[1.16, 1.19]
has_mediaTRUE	1.47 ***	0.54 ***	2.83 ***	5.18 ***
	[1.44, 1.51]	[0.53, 0.55]	[2.08, 3.85]	[4.64, 5.77]
has_URLTRUE	0.73 ***	0.38 ***	1.80 ***	5.04 ***
	[0.70, 0.75]	[0.37, 0.40]	[1.32, 2.44]	[4.53, 5.61]
followers_count	1.00 ***	1.00 ***		
	[1.00, 1.00]	[1.00, 1.00]		
is_retweetTRUE	1.54 ***	0.79 ***		
	[1.48, 1.60]	[0.76, 0.82]		
`Likes at Posting`			1.00 ***	1.00 ***
			[1.00, 1.00]	[1.00, 1.00]
N	143702	83527	300000	299999
AIC	489760.05	294339.53	1142251.76	1206259.31
BIC	489868.68	294442.19	1142357.88	1206365.43
Pseudo R2	0.19	0.17	0.15	0.10

*** $p < 0.001$; ** $p < 0.01$; * $p < 0.05$.

Table S2. VIFS for Study 1.

	Twitter		Facebook	
	Conservative	Liberal	Conservative	Liberal
	VIF	VIF	VIF	VIF
Democrat	1.02	1.02	1.04	1.02
Republican	1.01	1.01	1.03	1.02
NegativeAffect	1.3	1.24	1.41	1.32
PositiveAffect	1.11	1.07	1.07	1.06
MoralEmotional	1.37	1.29	1.46	1.38
has_media	1.09	1.11	55	370.06
has_URL	1.6	2.74	55.52	370.08
followers_count	1.02	1.01		
is_retweet	1.65	2.72		
Likes at Posting			1.29	1.01

Note. Variance Inflation Factors (VIFS) for study 1. It should be noted that some VIFs are extremely high. This is because, in some cases, these variables are almost perfectly inversely correlated (e.g., if a Facebook post did not have a URL, it had media). These high VIFS were for control variables, not for key variables. We ran the model without control variables and the results were similar (Table S3) and the results were less problematic (Table S5).

Table S3. Study 1 Regression Models Without Control Variables

	Twitter		Facebook	
	Liberal	Conservative	Liberal	Conservative
(Intercept)	13.50 ***	8.68 ***	80.90 ***	99.70 ***
	[13.44, 13.55]	[8.64, 8.72]	[80.40, 81.40]	[99.03, 100.37]
Democrat	1.04 ***	3.27 ***	0.97 ***	1.23 ***
	[1.04, 1.05]	[3.23, 3.32]	[0.97, 0.98]	[1.22, 1.24]
Republican	2.41 ***	1.26 ***	1.47 ***	1.27 ***
	[2.38, 2.43]	[1.25, 1.27]	[1.46, 1.48]	[1.26, 1.29]
NegativeAffect	1.36 ***	1.47 ***	1.04 ***	0.98 ***
	[1.35, 1.37]	[1.46, 1.48]	[1.03, 1.04]	[0.98, 0.99]
PositiveAffect	0.91 ***	1.00	0.89 ***	0.92 ***
	[0.91, 0.92]	[1.00, 1.01]	[0.89, 0.90]	[0.92, 0.93]
MoralEmotional	1.11 ***	1.07 ***	1.12 ***	1.18 ***
	[1.10, 1.12]	[1.06, 1.08]	[1.10, 1.13]	[1.17, 1.20]
N	747675	611292	300000	299999
AIC	3069655.63	2530345.65	1179454.34	1232001.33
BIC	3069736.30	2530424.91	1179528.62	1232075.61
Pseudo R2	0.08	0.09	0.03	0.02

*** $p < 0.001$; ** $p < 0.01$; * $p < 0.05$.

Table S4. VIFS for Study 1 Regression Without Control Variables

	Twitter		Facebook	
	Liberal	Conservative	Liberal	Conservative
	VIF	VIF	VIF	VIF
Democrat	1.01	1.01	1.01	1.02
Republican	1.01	1.01	1.01	1.02
NegativeAffect	1.24	1.29	1.32	1.41
PositiveAffect	1.06	1.08	1.06	1.07
MoralEmotional	1.29	1.37	1.38	1.46

Table S5. Study 1 Robustness Check (Cluster Robust Standard Errors)

	Twitter		Facebook	
	Liberal	Conservative	Liberal	Conservative
(Intercept)	38.04 ***	68.68 ***	41.96 ***	19.75 ***
	[36.52, 39.62]	[65.73, 71.76]	[30.12, 58.46]	[17.51, 22.29]
Democrat	1.10 ***	1.29 ***	1.00	1.35 ***
	[1.08, 1.12]	[1.26, 1.32]	[0.99, 1.00]	[1.34, 1.36]
Republican	1.46 ***	1.23 ***	1.57 ***	1.37 ***
	[1.43, 1.48]	[1.20, 1.26]	[1.55, 1.58]	[1.35, 1.38]
NegativeAffect	1.08 ***	1.08 ***	1.05 ***	0.98 ***
	[1.07, 1.09]	[1.06, 1.09]	[1.05, 1.06]	[0.98, 0.99]
PositiveAffect	0.89 ***	0.98 **	0.90 ***	0.94 ***
	[0.88, 0.90]	[0.97, 0.99]	[0.89, 0.90]	[0.93, 0.94]
MoralEmotional	1.17 ***	1.10 ***	1.10 ***	1.17 ***
	[1.16, 1.19]	[1.07, 1.13]	[1.09, 1.11]	[1.15, 1.20]
has_mediaTRUE	1.47 ***	0.54 ***	2.83 ***	5.18 ***
	[1.44, 1.51]	[0.53, 0.55]	[2.03, 3.94]	[4.58, 5.85]
has_URLTRUE	0.73 ***	0.38 ***	1.80 ***	5.04 ***
	[0.70, 0.76]	[0.37, 0.40]	[1.29, 2.50]	[4.47, 5.69]
followers_count	1.00 ***	1.00 ***		
	[1.00, 1.00]	[1.00, 1.00]		
is_retweetTRUE	1.54 ***	0.79 ***		
	[1.48, 1.61]	[0.76, 0.82]		
`Likes at Posting`			1.00 ***	1.00 ***
			[1.00, 1.00]	[1.00, 1.00]
N	143702	83527	300000	299999
AIC	489760.05	294339.53	1142251.76	1206259.31
BIC	489868.68	294442.19	1142357.88	1206365.43
Pseudo R2	0.19	0.17	0.15	0.10

*** $p < 0.001$; ** $p < 0.01$; * $p < 0.05$.

Table S5. Study 1 Relative Importance Analysis

	Twitter		Facebook	
	Conservative	Liberal	Conservative	Liberal
Democrat	**0.00734**	0.0005	**0.01082**	0.000076
Republican	0.00439	**0.0095**	0.01014	**0.026482**
NegativeAffect	0.0023	0.0027	0.00032	0.001931
PositiveAffect	0.00042	0.0032	0.00074	0.004462
MoralEmotional	0.00149	0.0026	0.00204	0.001477
has_media	0.02948	0.0073	0.00763	0.010908
has_URL	0.03801	0.0204		
followers_count	0.07837	0.1384		
Likes at Posting			0.06433	0.098183

Note. "lmg" (or estimated R^2) values are shown for each regression model

Table S6. Liberal Media Reactions Regression Analysis

	Shares	Comments	Likes	Love	Wow	Haha	Sad	Angry	Retweet	Favorites
(Intercept)	41.96 ***	130.60 ***	191.46 ***	12.93 ***	12.59 ***	13.10 ***	12.40 ***	10.44 ***	38.04 ***	80.08 ***
	[30.85, 57.08]	[97.25, 175.37]	[146.85, 249.63]	[9.18, 18.21]	[9.32, 17.00]	[9.60, 17.87]	[8.43, 18.24]	[6.97, 15.64]	[36.71, 39.42]	[77.23, 83.02]
Democrat	1.00	1.57 ***	1.31 ***	1.66 ***	0.91 ***	1.72 ***	0.87 ***	1.15 ***	1.10 ***	1.25 ***
	[0.99, 1.00]	[1.56, 1.58]	[1.30, 1.32]	[1.64, 1.68]	[0.90, 0.92]	[1.71, 1.74]	[0.86, 0.88]	[1.13, 1.16]	[1.09, 1.12]	[1.23, 1.27]
Republican	1.57 ***	2.23 ***	1.45 ***	1.44 ***	1.54 ***	2.92 ***	1.35 ***	3.33 ***	1.46 ***	1.22 ***
	[1.55, 1.58]	[2.21, 2.24]	[1.44, 1.46]	[1.42, 1.45]	[1.52, 1.55]	[2.90, 2.95]	[1.33, 1.36]	[3.30, 3.37]	[1.44, 1.48]	[1.20, 1.24]
NegativeAffect	1.05 ***	1.05 ***	0.99 ***	0.88 ***	1.06 ***	1.01 ***	1.31 ***	1.18 ***	1.08 ***	1.04 ***
	[1.05, 1.06]	[1.05, 1.06]	[0.98, 0.99]	[0.87, 0.88]	[1.05, 1.06]	[1.01, 1.02]	[1.30, 1.32]	[1.17, 1.18]	[1.07, 1.09]	[1.03, 1.05]
PositiveAffect	0.90 ***	0.86 ***	0.98 ***	1.12 ***	0.79 ***	0.92 ***	0.77 ***	0.78 ***	0.89 ***	0.95 ***
	[0.89, 0.90]	[0.86, 0.87]	[0.98, 0.99]	[1.11, 1.13]	[0.79, 0.79]	[0.92, 0.93]	[0.76, 0.77]	[0.77, 0.78]	[0.88, 0.90]	[0.95, 0.96]
MoralEmotional	1.10 ***	1.07 ***	1.05 ***	1.05 ***	1.10 ***	0.91 ***	1.22 ***	1.23 ***	1.17 ***	1.13 ***
	[1.09, 1.11]	[1.06, 1.08]	[1.04, 1.05]	[1.04, 1.06]	[1.09, 1.11]	[0.90, 0.92]	[1.20, 1.23]	[1.21, 1.25]	[1.16, 1.19]	[1.11, 1.15]
has_URLTRUE	1.80 ***	0.78	1.00	0.79	1.09	0.97	1.04	1.14	0.73 ***	0.80 ***
	[1.32, 2.44]	[0.58, 1.05]	[0.76, 1.30]	[0.56, 1.11]	[0.80, 1.47]	[0.71, 1.33]	[0.71, 1.53]	[0.76, 1.71]	[0.70, 0.75]	[0.77, 0.83]
has_mediaTRUE	2.83 ***	1.30	1.68 ***	2.33 ***	1.30	1.27	1.16	1.12	1.47 ***	1.66 ***
	[2.08, 3.85]	[0.97, 1.74]	[1.29, 2.19]	[1.66, 3.29]	[0.96, 1.76]	[0.93, 1.73]	[0.79, 1.70]	[0.75, 1.68]	[1.44, 1.51]	[1.62, 1.70]
`Likes at Posting`	1.00 ***	1.00 ***	1.00 ***	1.00 ***	1.00 ***	1.00 ***	1.00 ***	1.00 ***		
	[1.00, 1.00]	[1.00, 1.00]	[1.00, 1.00]	[1.00, 1.00]	[1.00, 1.00]	[1.00, 1.00]	[1.00, 1.00]	[1.00, 1.00]		
followers_count									1.00 ***	1.00 ***
									[1.00, 1.00]	[1.00, 1.00]
is_retweetTRUE									1.54 ***	0.01 ***
									[1.48, 1.60]	[0.01, 0.01]
N	300000	300000	300000	300000	300000	300000	300000	300000	143702	143702
AIC	1142251.76	1116558.15	1053241.80	1206829.92	1127482.48	1148172.38	1277786.18	1305894.87	489760.05	494589.33
BIC	1142357.88	1116664.26	1053347.91	1206936.04	1127588.59	1148278.49	1277892.30	1306000.98	489868.68	494697.97
Pseudo R2	0.15	0.26	0.22	0.19	0.17	0.26	0.11	0.17	0.19	0.54

Table S7. Conservative Media Reactions Regression Analysis

	Shares	Comments	Likes	Love	Wow	Haha	Sad	Angry	Retweet	Favorites
(Intercept)	19.75 ***	28.89 ***	71.18 ***	11.15 ***	3.26 ***	3.98 ***	3.15 ***	3.17 ***	38.04 ***	80.08 ***
	[17.74, 22.00]	[26.09, 32.00]	[64.79, 78.19]	[9.94, 12.51]	[2.97, 3.59]	[3.53, 4.49]	[2.82, 3.52]	[2.76, 3.65]	[36.71, 39.42]	[77.23, 83.02]
Democrat	1.35 ***	1.59 ***	1.18 ***	1.08 ***	1.27 ***	2.47 ***	1.16 ***	1.83 ***	1.10 ***	1.25 ***
	[1.34, 1.36]	[1.58, 1.60]	[1.17, 1.19]	[1.06, 1.09]	[1.26, 1.28]	[2.45, 2.50]	[1.15, 1.17]	[1.81, 1.85]	[1.09, 1.12]	[1.23, 1.27]
Republican	1.37 ***	1.81 ***	1.77 ***	2.26 ***	0.99	1.59 ***	0.96 ***	1.47 ***	1.46 ***	1.22 ***
	[1.35, 1.38]	[1.80, 1.83]	[1.76, 1.79]	[2.24, 2.29]	[0.98, 1.00]	[1.57, 1.61]	[0.95, 0.97]	[1.45, 1.49]	[1.44, 1.48]	[1.20, 1.24]
NegativeAffect	0.98 ***	0.95 ***	0.90 ***	0.79 ***	1.01 **	0.93 ***	1.21 ***	1.09 ***	1.08 ***	1.04 ***
	[0.98, 0.99]	[0.94, 0.95]	[0.90, 0.91]	[0.79, 0.80]	[1.00, 1.02]	[0.92, 0.94]	[1.20, 1.22]	[1.08, 1.10]	[1.07, 1.09]	[1.03, 1.05]
PositiveAffect	0.94 ***	0.89 ***	1.05 ***	1.21 ***	0.83 ***	0.92 ***	0.82 ***	0.75 ***	0.89 ***	0.95 ***
	[0.93, 0.94]	[0.88, 0.89]	[1.04, 1.06]	[1.20, 1.21]	[0.82, 0.83]	[0.91, 0.92]	[0.81, 0.82]	[0.74, 0.75]	[0.88, 0.90]	[0.95, 0.96]
MoralEmotional	1.17 ***	1.13 ***	1.12 ***	1.14 ***	1.10 ***	0.91 ***	1.20 ***	1.27 ***	1.17 ***	1.13 ***
	[1.16, 1.19]	[1.12, 1.14]	[1.11, 1.13]	[1.13, 1.15]	[1.09, 1.11]	[0.89, 0.92]	[1.18, 1.21]	[1.25, 1.29]	[1.16, 1.19]	[1.11, 1.15]
has_URLTRUE	5.04 ***	5.59 ***	3.23 ***	0.97	6.02 ***	7.45 ***	3.48 ***	6.98 ***	0.73 ***	0.80 ***
	[4.53, 5.61]	[5.04, 6.19]	[2.94, 3.55]	[0.86, 1.08]	[5.47, 6.62]	[6.60, 8.41]	[3.12, 3.89]	[6.06, 8.03]	[0.70, 0.75]	[0.77, 0.83]
has_mediaTRUE	5.18 ***	5.14 ***	4.04 ***	1.75 ***	3.64 ***	6.75 ***	2.13 ***	4.20 ***	1.47 ***	1.66 ***
	[4.64, 5.77]	[4.63, 5.70]	[3.68, 4.45]	[1.56, 1.97]	[3.30, 4.00]	[5.98, 7.63]	[1.91, 2.38]	[3.64, 4.83]	[1.44, 1.51]	[1.62, 1.70]
`Likes at Posting`	1.00 ***	1.00 ***	1.00 ***	1.00 ***	1.00 ***	1.00 ***	1.00 ***	1.00 ***		
	[1.00, 1.00]	[1.00, 1.00]	[1.00, 1.00]	[1.00, 1.00]	[1.00, 1.00]	[1.00, 1.00]	[1.00, 1.00]	[1.00, 1.00]		
followers_count									1.00 ***	1.00 ***
									[1.00, 1.00]	[1.00, 1.00]
is_retweetTRUE									1.54 ***	0.01 ***
									[1.48, 1.60]	[0.01, 0.01]
N	299999	299999	299999	299999	299999	299999	299999	299999	143702	143702
AIC	1206259.31	1175256.73	1125262.30	1246255.47	1132021.07	1275974.00	1219208.80	1365334.17	489760.05	494589.33
BIC	1206365.43	1175362.84	1125368.41	1246361.59	1132127.18	1276080.12	1219314.91	1365440.28	489868.68	494697.97
Pseudo R2	0.10	0.18	0.17	0.18	0.14	0.15	0.13	0.08	0.19	0.54

Table S8. Study 1 Descriptive Statistics

	Conservative Twitter		Liberal Twitter		Conservative Facebook		Liberal Facebook	
Variable	*M*	*SD*	*M*	*SD*	*M*	*SD*	*M*	*SD*
1. Democrat	0.18	0.50	0.13	0.45	0.20	0.68	0.31	0.74
2. Republican	0.17	0.44	0.15	0.42	0.27	0.69	0.29	0.65
3. Positive Affect	0.42	0.73	0.55	0.82	0.81	1.14	0.67	0.99
4. Negative Affect	0.41	0.70	0.52	0.79	0.82	1.13	0.76	1.04
5. Moral Emotional	0.18	0.45	0.21	0.49	0.33	0.70	0.31	0.67

Note. Means and standard deviations for each of the language categories in each dataset.

Table S9. Liberal Media Facebook Reactions

Variable	*M*	*SD*	1	2	3	4	5	6	7
1. Shares	491.17	3984.80							
2. Likes	864.71	4124.87	.63**<br>[.62, .63]						
3. Comments	410.85	1009.18	.43**<br>[.43, .44]	.49**<br>[.48, .49]					
4. Love	169.58	1290.41	.32**<br>[.32, .32]	.79**<br>[.78, .79]	.32**<br>[.31, .32]				
5. Wow	82.22	686.84	.51**<br>[.50, .51]	.45**<br>[.44, .45]	.33**<br>[.32, .33]	.17**<br>[.16, .17]			
6. Haha	106.47	571.40	.16**<br>[.16, .17]	.22**<br>[.21, .22]	.43**<br>[.43, .44]	.14**<br>[.13, .14]	.12**<br>[.12, .13]		
7. Sad	181.40	1343.43	.42**<br>[.42, .42]	.28**<br>[.28, .29]	.26**<br>[.25, .26]	.10**<br>[.09, .10]	.23**<br>[.22, .23]	.02**<br>[.01, .02]	
8. Angry	231.56	1123.14	.22**<br>[.22, .23]	.07**<br>[.07, .08]	.46**<br>[.46, .47]	.00**<br>[.00, .01]	.18**<br>[.17, .18]	.14**<br>[.14, .15]	.13**<br>[.13, .14]

Note. M and *SD* are used to represent mean and standard deviation, respectively. Values in square brackets indicate the 95% confidence interval for each correlation. * indicates $p < .05$. ** indicates $p < .01$.

Table S10. Conservative Media Correlations and Descriptive Statistics

Variable	*M*	*SD*	1	2	3	4	5	6	7
1. Shares	755.51	4580.27							
2. Likes	1491.57	7194.08	.55**<br>[.55, .55]						
3. Comments	866.29	3159.10	.34**<br>[.34, .34]	.38**<br>[.37, .38]					
4. Love	264.84	2143.31	.49**<br>[.49, .49]	.84**<br>[.84, .84]	.31**<br>[.31, .31]				
5. Wow	89.36	435.82	.48**<br>[.48, .48]	.33**<br>[.32, .33]	.29**<br>[.28, .29]	.27**<br>[.27, .28]			
6. Haha	367.68	2074.96	.26**<br>[.26, .27]	.17**<br>[.17, .17]	.55**<br>[.55, .55]	.10**<br>[.10, .11]	.19**<br>[.19, .19]		
7. Sad	138.69	1447.45	.26**<br>[.26, .27]	.09**<br>[.09, .10]	.13**<br>[.13, .13]	.06**<br>[.06, .07]	.18**<br>[.18, .18]	.01**<br>[.01, .02]	
8. Angry	414.01	2054.33	.21**<br>[.21, .22]	.05**<br>[.05, .06]	.60**<br>[.60, .61]	.01**<br>[.01, .01]	.29**<br>[.29, .30]	.29**<br>[.29, .30]	.14**<br>[.14, .15]

Note. M and *SD* are used to represent mean and standard deviation, respectively. Values in square brackets indicate the 95% confidence interval for each correlation. * indicates $p < .05$. ** indicates $p < .01$

Table S11. Study 2 Regression Models

	Facebook		Twitter	
	Liberal	Conservative	Liberal	Conservative
(Intercept)	8.79 ***	7.83 ***	9.70 ***	6.25 ***
	[8.65, 8.94]	[7.71, 7.95]	[9.63, 9.78]	[6.20, 6.30]
Democrat	1.02 ***	1.65 ***	0.75 ***	2.80 ***
	[1.01, 1.03]	[1.64, 1.67]	[0.75, 0.75]	[2.77, 2.84]
Republican	1.58 ***	1.20 ***	2.13 ***	0.85 ***
	[1.57, 1.59]	[1.19, 1.20]	[2.11, 2.15]	[0.84, 0.85]
NegativeAffect	1.14 ***	1.12 ***	1.33 ***	1.45 ***
	[1.13, 1.14]	[1.11, 1.12]	[1.33, 1.34]	[1.44, 1.45]
PositiveAffect	0.96 ***	0.98 ***	0.95 ***	1.04 ***
	[0.95, 0.96]	[0.97, 0.98]	[0.95, 0.95]	[1.04, 1.05]
MoralEmotional	1.06 ***	1.05 ***	1.10 ***	1.06 ***
	[1.05, 1.06]	[1.04, 1.05]	[1.10, 1.11]	[1.05, 1.07]
has_URLTRUE	1.24 ***	1.00	0.95 ***	0.83 ***
	[1.21, 1.26]	[0.98, 1.01]	[0.94, 0.96]	[0.82, 0.83]
has_mediaTRUE	1.09 ***	0.92 ***	1.08 ***	1.22 ***
	[1.08, 1.11]	[0.91, 0.94]	[1.07, 1.09]	[1.20, 1.23]
`Likes at Posting`	1.00 ***	1.00 ***		
	[1.00, 1.00]	[1.00, 1.00]		
followers_count			1.00 ***	1.00 ***
			[1.00, 1.00]	[1.00, 1.00]
is_retweetTRUE			5.10 ***	5.36 ***
			[5.04, 5.16]	[5.30, 5.42]
N	354814	410313	747675	611292
AIC	1253213.12	1474731.77	2861220.18	2343833.57
BIC	1253320.92	1474841.02	2861346.95	2343958.13
Pseudo R2	0.33	0.13	0.30	0.33

*** $p < 0.001$; ** $p < 0.01$; * $p < 0.05$.

Table S12. VIFS to Study 2

	Twitter		Facebook	
	Conservative	Liberal	Conservative	Liberal
	VIF	VIF	VIF	VIF
Democrat	1.05	1.13	1.09	1.02
Republican	1.14	1.03	1.08	1.1
NegativeAffect	1.23	1.32	1.63	1.52
PositiveAffect	1.24	1.22	1.41	1.36
MoralEmotional	1.4	1.47	1.95	1.8
has_media	1.33	1.33	3.27	3.67
has_URL	1.38	1.37	3.32	3.71
followers_count	1.01	1.01		
is_retweet	1.34	1.42		
Likes at Posting			1	1.01

Note. Variance Inflation Factors (VIFS) for study 2.

Table S13. Study 2 Models Without Control Variables

	Twitter		Facebook	
	Liberal	Conservative	Liberal	Conservative
(Intercept)	31.36 ***	23.97 ***	80.90 ***	99.70 ***
	[31.13, 31.60]	[23.72, 24.22]	[80.40, 81.40]	[99.03, 100.37]
Democrat	1.03 **	1.29 ***	0.97 ***	1.23 ***
	[1.01, 1.05]	[1.26, 1.32]	[0.97, 0.98]	[1.22, 1.24]
Republican	1.35 ***	1.26 ***	1.47 ***	1.27 ***
	[1.32, 1.37]	[1.23, 1.29]	[1.46, 1.48]	[1.26, 1.29]
NegativeAffect	1.08 ***	1.10 ***	1.04 ***	0.98 ***
	[1.06, 1.09]	[1.08, 1.12]	[1.03, 1.04]	[0.98, 0.99]
PositiveAffect	0.89 ***	1.04 ***	0.89 ***	0.92 ***
	[0.89, 0.90]	[1.02, 1.05]	[0.89, 0.90]	[0.92, 0.93]
MoralEmotional	1.17 ***	1.11 ***	1.12 ***	1.18 ***
	[1.15, 1.19]	[1.08, 1.14]	[1.10, 1.13]	[1.17, 1.20]
N	143702	83527	300000	299999
AIC	517325.84	307824.60	1179454.34	1232001.33
BIC	517394.97	307889.93	1179528.62	1232075.61
Pseudo R2	0.02	0.02	0.03	0.02

*** $p < 0.001$; ** $p < 0.01$; * $p < 0.05$.

Table S14. VIFS for Study 2 Models Without Control Variables

	Twitter		Facebook	
	Liberal	Conservative	Liberal	Conservative
	VIF	VIF	VIF	VIF
Democrat	1.01	1.04	1.02	1.09
Republican	1.02	1.03	1.08	1.06
NegativeAffect	1.31	1.22	1.5	1.6
PositiveAffect	1.2	1.2	1.36	1.41
MoralEmotional	1.47	1.4	1.81	1.95

Table S15. Study 2 With Cluster Robust Standard Errors

	Facebook		Twitter	
	Liberal	Conservative	Liberal	Conservative
(Intercept)	8.79 ***	7.83 ***	9.70 ***	6.25 ***
	[8.65, 8.94]	[7.70, 7.96]	[9.63, 9.78]	[6.20, 6.30]
Democrat	1.02 ***	1.65 ***	0.75 ***	2.80 ***
	[1.01, 1.03]	[1.62, 1.68]	[0.74, 0.76]	[2.75, 2.86]
Republican	1.58 ***	1.20 ***	2.13 ***	0.85 ***
	[1.56, 1.59]	[1.18, 1.21]	[2.10, 2.15]	[0.84, 0.86]
NegativeAffect	1.14 ***	1.12 ***	1.33 ***	1.45 ***
	[1.13, 1.14]	[1.11, 1.12]	[1.33, 1.34]	[1.43, 1.46]
PositiveAffect	0.96 ***	0.98 ***	0.95 ***	1.04 ***
	[0.95, 0.96]	[0.97, 0.98]	[0.95, 0.95]	[1.04, 1.05]
MoralEmotional	1.06 ***	1.05 ***	1.10 ***	1.06 ***
	[1.05, 1.06]	[1.04, 1.05]	[1.10, 1.11]	[1.05, 1.07]
has_mediaTRUE	1.09 ***	0.92 ***	1.08 ***	1.22 ***
	[1.07, 1.11]	[0.91, 0.94]	[1.07, 1.09]	[1.21, 1.23]
has_URLTRUE	1.24 ***	1.00	0.95 ***	0.83 ***
	[1.21, 1.26]	[0.98, 1.02]	[0.94, 0.96]	[0.82, 0.83]
followers_count			1.00 ***	1.00 ***
			[1.00, 1.00]	[1.00, 1.00]
is_retweetTRUE			5.10 ***	5.36 ***
			[5.03, 5.18]	[5.28, 5.44]
`Likes at Posting`	1.00 ***	1.00 ***		
	[1.00, 1.00]	[1.00, 1.00]		
N	354814	410313	747675	611292
AIC	1253213.12	1474731.77	2861220.18	2343833.57
BIC	1253320.92	1474841.02	2861346.95	2343958.13
Pseudo R2	0.33	0.13	0.30	0.33

*** $p < 0.001$; ** $p < 0.01$; * $p < 0.05$.

Table S16. Study 2 Relative Importance Analysis

	Twitter		Facebook	
	Conservative	Liberal	Conservative	Liberal
	Img	Img	Img	Img
Democrat	**0.046435128**	0.003990291	**0.037326404**	0.000695989
Republican	0.004320336	**0.035396725**	0.010754976	**0.045910789**
NegativeAffect	0.020092764	0.021551454	0.018937441	0.022214026
PositiveAffect	0.000413394	0.002722643	0.001024292	0.002541912
MoralEmotional	0.003008898	0.004656238	0.005519233	0.006538567
has_media	0.002206309	0.00426283	0.000992487	0.003629467
has_URL	0.01654347	0.006841593	0.002193025	0.002415596
followers_count	0.131899268	0.136219852		
is_retweet	0.10321281	0.08377927		
Likes at Posting			0.048458365	0.244020805

Table S17. Study 2 Conservative Congress Facebook Reactions

	Shares	Comments	Likes	Loves	Wow	Haha	Sad	Angry	Retweet	Favorite
(Intercept)	7.83 ***	31.95 ***	64.91 ***	4.56 ***	1.56 ***	2.36 ***	2.03 ***	3.08 ***	6.25 ***	12.08 ***
	[7.71, 7.95]	[31.38, 32.52]	[63.96, 65.87]	[4.50, 4.63]	[1.55, 1.57]	[2.33, 2.38]	[2.01, 2.04]	[3.04, 3.13]	[6.20, 6.30]	[11.99, 12.18]
Democrat	1.65 ***	1.58 ***	1.32 ***	1.13 ***	1.31 ***	1.43 ***	1.26 ***	1.68 ***	2.80 ***	1.93 ***
	[1.64, 1.67]	[1.56, 1.60]	[1.31, 1.33]	[1.12, 1.14]	[1.30, 1.31]	[1.42, 1.43]	[1.25, 1.27]	[1.67, 1.69]	[2.77, 2.84]	[1.91, 1.95]
Republican	1.20 ***	1.41 ***	1.26 ***	1.32 ***	1.05 ***	1.28 ***	1.04 ***	1.25 ***	0.85 ***	1.18 ***
	[1.19, 1.20]	[1.40, 1.42]	[1.25, 1.27]	[1.31, 1.33]	[1.04, 1.05]	[1.27, 1.29]	[1.04, 1.04]	[1.24, 1.26]	[0.84, 0.85]	[1.17, 1.19]
NegativeAffect	1.12 ***	1.10 ***	1.05 ***	1.00	1.04 ***	1.00	1.10 ***	1.06 ***	1.45 ***	1.37 ***
	[1.11, 1.12]	[1.09, 1.10]	[1.05, 1.05]	[0.99, 1.00]	[1.03, 1.04]	[1.00, 1.00]	[1.10, 1.10]	[1.06, 1.06]	[1.44, 1.45]	[1.36, 1.38]
PositiveAffect	0.98 ***	0.99 ***	1.01 ***	1.02 ***	0.98 ***	0.98 ***	0.97 ***	0.96 ***	1.04 ***	1.16 ***
	[0.97, 0.98]	[0.99, 0.99]	[1.01, 1.02]	[1.02, 1.02]	[0.98, 0.98]	[0.98, 0.98]	[0.97, 0.97]	[0.96, 0.96]	[1.04, 1.05]	[1.16, 1.16]
MoralEmotional	1.05 ***	1.03 ***	1.02 ***	1.05 ***	1.00	1.00	1.03 ***	1.02 ***	1.06 ***	1.03 ***
	[1.04, 1.05]	[1.03, 1.04]	[1.02, 1.03]	[1.04, 1.05]	[1.00, 1.00]	[0.99, 1.00]	[1.03, 1.04]	[1.02, 1.03]	[1.05, 1.07]	[1.03, 1.04]
has_URLTRUE	1.00	0.64 ***	0.69 ***	0.69 ***	0.99 **	0.82 ***	0.77 ***	0.79 ***	0.83 ***	0.81 ***
	[0.98, 1.01]	[0.63, 0.65]	[0.68, 0.70]	[0.68, 0.71]	[0.98, 1.00]	[0.81, 0.83]	[0.77, 0.78]	[0.78, 0.81]	[0.82, 0.83]	[0.80, 0.82]
has_mediaTRUE	0.92 ***	0.65 ***	0.95 ***	0.96 ***	0.92 ***	0.84 ***	0.73 ***	0.74 ***	1.22 ***	1.52 ***
	[0.91, 0.94]	[0.64, 0.67]	[0.94, 0.97]	[0.95, 0.98]	[0.91, 0.92]	[0.83, 0.84]	[0.73, 0.74]	[0.73, 0.75]	[1.20, 1.23]	[1.50, 1.53]
`Likes at Posting`	1.00 ***	1.00 ***	1.00 ***	1.00 ***	1.00 ***	1.00 ***	1.00 ***	1.00 ***		
	[1.00, 1.00]	[1.00, 1.00]	[1.00, 1.00]	[1.00, 1.00]	[1.00, 1.00]	[1.00, 1.00]	[1.00, 1.00]	[1.00, 1.00]		
is_retweetTRUE									5.36 ***	0.07 ***
									[5.30, 5.42]	[0.07, 0.07]
followers_count									1.00 ***	1.00 ***
									[1.00, 1.00]	[1.00, 1.00]
N	410313	410313	410313	410313	410313	410313	410313	410313	611292	611292
AIC	1474731.77	1608010.64	1457229.58	1422293.10	900551.43	1183686.06	1069002.13	1375557.79	2343833.57	2265816.97
BIC	1474841.02	1608119.89	1457338.82	1422402.35	900660.68	1183795.31	1069111.38	1375667.04	2343958.13	2265941.53
Pseudo R2	0.13	0.11	0.11	0.09	0.13	0.13	0.12	0.13	0.33	0.43

Table S18. Study 2 Liberal Congress Facebook Reactions

	Shares	Comments	Likes	Loves	Wow	Haha	Sad	Angry	Retweet	Favorite
(Intercept)	8.79 ***	26.67 ***	81.16 ***	7.74 ***	1.73 ***	2.76 ***	2.73 ***	2.87 ***	9.70 ***	25.87 ***
	[8.65, 8.94]	[26.14, 27.21]	[79.87, 82.47]	[7.61, 7.88]	[1.71, 1.74]	[2.73, 2.80]	[2.69, 2.77]	[2.82, 2.91]	[9.63, 9.78]	[25.69, 26.05]
Democrat	1.02 ***	1.16 ***	1.12 ***	1.23 ***	0.97 ***	1.15 ***	0.89 ***	0.93 ***	0.75 ***	1.04 ***
	[1.01, 1.03]	[1.15, 1.17]	[1.11, 1.13]	[1.22, 1.24]	[0.97, 0.98]	[1.14, 1.16]	[0.89, 0.90]	[0.92, 0.93]	[0.75, 0.75]	[1.04, 1.05]
Republican	1.58 ***	1.75 ***	1.28 ***	1.01	1.32 ***	1.45 ***	1.42 ***	2.24 ***	2.13 ***	1.69 ***
	[1.57, 1.59]	[1.74, 1.76]	[1.27, 1.29]	[1.00, 1.01]	[1.32, 1.33]	[1.44, 1.45]	[1.41, 1.42]	[2.22, 2.25]	[2.11, 2.15]	[1.68, 1.70]
NegativeAffect	1.14 ***	1.17 ***	1.08 ***	1.01 ***	1.07 ***	1.04 ***	1.26 ***	1.17 ***	1.33 ***	1.30 ***
	[1.13, 1.14]	[1.17, 1.18]	[1.07, 1.08]	[1.01, 1.01]	[1.07, 1.07]	[1.04, 1.04]	[1.25, 1.26]	[1.17, 1.17]	[1.33, 1.34]	[1.29, 1.30]
PositiveAffect	0.96 ***	0.94 ***	1.02 ***	1.03 ***	0.96 ***	0.95 ***	0.92 ***	0.90 ***	0.95 ***	1.05 ***
	[0.95, 0.96]	[0.94, 0.95]	[1.02, 1.02]	[1.03, 1.03]	[0.96, 0.97]	[0.95, 0.95]	[0.92, 0.92]	[0.90, 0.90]	[0.95, 0.95]	[1.05, 1.05]
MoralEmotional	1.06 ***	1.09 ***	1.04 ***	1.05 ***	1.00	1.02 ***	1.05 ***	1.06 ***	1.10 ***	1.05 ***
	[1.05, 1.06]	[1.08, 1.10]	[1.03, 1.04]	[1.04, 1.05]	[1.00, 1.00]	[1.02, 1.03]	[1.04, 1.05]	[1.06, 1.07]	[1.10, 1.11]	[1.05, 1.06]
has_URLTRUE	1.24 ***	0.70 ***	0.66 ***	0.61 ***	1.24 ***	0.81 ***	1.04 ***	1.28 ***	0.95 ***	0.87 ***
	[1.21, 1.26]	[0.68, 0.71]	[0.65, 0.67]	[0.60, 0.62]	[1.23, 1.26]	[0.80, 0.82]	[1.03, 1.06]	[1.26, 1.30]	[0.94, 0.96]	[0.86, 0.87]
has_mediaTRUE	1.09 ***	0.66 ***	0.85 ***	0.94 ***	0.99	0.82 ***	0.74 ***	0.87 ***	1.08 ***	1.15 ***
	[1.08, 1.11]	[0.65, 0.68]	[0.84, 0.87]	[0.92, 0.96]	[0.98, 1.00]	[0.81, 0.84]	[0.73, 0.75]	[0.85, 0.88]	[1.07, 1.09]	[1.14, 1.16]
`Likes at Posting`	1.00 ***	1.00 ***	1.00 ***	1.00 ***	1.00 ***	1.00 ***	1.00 ***	1.00 ***		
	[1.00, 1.00]	[1.00, 1.00]	[1.00, 1.00]	[1.00, 1.00]	[1.00, 1.00]	[1.00, 1.00]	[1.00, 1.00]	[1.00, 1.00]		
is_retweetTRUE									5.10 ***	0.04 ***
									[5.04, 5.16]	[0.04, 0.04]
followers_count									1.00 ***	1.00 ***
									[1.00, 1.00]	[1.00, 1.00]
N	354814	354814	354814	354814	354814	354814	354814	354814	747675	747675
AIC	1253213.12	1391799.69	1228236.29	1266431.32	878864.12	1059362.91	1147845.85	1222456.91	2861220.18	2760433.97
BIC	1253320.92	1391907.49	1228344.08	1266539.11	878971.92	1059470.70	1147953.64	1222564.70	2861346.95	2760560.74
Pseudo R2	0.33	0.22	0.25	0.17	0.42	0.23	0.36	0.39	0.30	0.51

Table S19. Liberal Congress Facebook Reactions

Variable	*M*	*SD*	1	2	3	4	5	6	7
1. Shares	134.14	1590.63							
2. Likes	333.68	1722.16	.60**<br>[.59, .60]						
3. Comments	124.52	506.20	.45**<br>[.44, .45]	.57**<br>[.57, .58]					
4. Love	41.35	325.31	.43**<br>[.43, .43]	.80**<br>[.80, .81]	.48**<br>[.48, .49]				
5. Wow	7.59	80.29	.52**<br>[.52, .52]	.41**<br>[.41, .42]	.38**<br>[.38, .38]	.15**<br>[.15, .16]			
6. Haha	8.46	88.31	.24**<br>[.24, .24]	.30**<br>[.30, .31]	.40**<br>[.40, .40]	.21**<br>[.21, .21]	.24**<br>[.24, .25]		
7. Sad	28.75	272.60	.38**<br>[.38, .38]	.35**<br>[.35, .36]	.34**<br>[.34, .34]	.12**<br>[.12, .13]	.40**<br>[.40, .40]	.09**<br>[.09, .10]	
8. Angry	67.91	672.91	.41**<br>[.41, .42]	.31**<br>[.30, .31]	.43**<br>[.43, .43]	.04**<br>[.04, .05]	.63**<br>[.63, .63]	.20**<br>[.20, .20]	.45**<br>[.45, .45]

Note. M and *SD* are used to represent mean and standard deviation, respectively. Values in square brackets indicate the 95% confidence interval for each correlation. * indicates $p < .05$. ** indicates $p < .01$.

Table S20. Conservative Congress Facebook Reactions

Variable	*M*	*SD*	1	2	3	4	5	6	7
1. Shares	67.82	5292.50							
2. Likes	241.78	1659.19	.67**<br>[.66, .67]						
3. Comments	97.68	512.71	.41**<br>[.41, .42]	.54**<br>[.54, .54]					
4. Love	17.66	153.78	.12**<br>[.11, .12]	.44**<br>[.44, .45]	.35**<br>[.35, .35]				
5. Wow	2.09	35.43	.73**<br>[.73, .73]	.55**<br>[.55, .55]	.46**<br>[.46, .46]	.17**<br>[.17, .17]			
6. Haha	4.69	45.48	.06**<br>[.06, .06]	.18**<br>[.18, .18]	.34**<br>[.34, .34]	.23**<br>[.23, .23]	.19**<br>[.19, .19]		
7. Sad	5.07	109.59	.07**<br>[.06, .07]	.12**<br>[.12, .12]	.21**<br>[.21, .21]	.09**<br>[.09, .10]	.29**<br>[.29, .29]	.04**<br>[.04, .04]	
8. Angry	15.60	212.02	.47**<br>[.47, .47]	.38**<br>[.38, .39]	.64**<br>[.64, .64]	.13**<br>[.13, .13]	.67**<br>[.67, .67]	.22**<br>[.22, .23]	.22**<br>[.21, .22]

Note. M and *SD* are used to represent mean and standard deviation, respectively. Values in square brackets indicate the 95% confidence interval for each correlation. * indicates $p < .05$. ** indicates $p < .01$.

Table S21. Descriptive Statistics – Congress

	Conservative Twitter		Liberal Twitter		Conservative Congress		Liberal Congress	
Variable	*M*	*SD*	*M*	*SD*	*M*	*SD*	*M*	*SD*
1. Democrat	0.1	0.38	0.21	0.66	0.13	0.54	0.21	0.58
2. Republican	0.23	0.63	0.15	0.44	0.24	0.67	0.27	0.72
3. PositiveAffect	1.16	1.28	1.19	1.28	2.48	2.78	2.31	2.46
4. NegativeAffect	0.35	0.72	0.54	0.88	0.79	1.66	0.98	1.61
5. MoralEmotional	0.29	0.6	0.42	0.72	0.65	1.22	0.78	1.25

Table S22. Meta-Analyzed Effect Sizes (Facebook Reactions)

name	estimate	conf.low	conf.high	model
Shares	1.53	1.41	1.66	Outgroup
Comments	1.77	1.51	2.08	Outgroup
Likes	1.30	1.20	1.42	Outgroup
Love	1.15	0.99	1.33	Outgroup
Haha	1.96	1.40	2.75	Outgroup
Wow	1.36	1.28	1.44	Outgroup
Sad	1.29	1.19	1.40	Outgroup
Angry	2.19	1.68	2.84	Outgroup
Retweets	1.83	1.35	2.49	Outgroup
Favorites	1.49	1.22	1.81	Outgroup
Shares	1.14	0.99	1.30	Ingroup
Comments	1.47	1.24	1.74	Ingroup
Likes	1.34	1.13	1.60	Ingroup
Love	1.57	1.24	2.00	Ingroup
Haha	1.42	1.20	1.68	Ingroup
Wow	0.98	0.92	1.04	Ingroup
Sad	0.94	0.86	1.03	Ingroup
Angry	1.18	0.99	1.42	Ingroup
Retweets	0.96	0.81	1.14	Ingroup
Favorites	1.20	1.09	1.32	Ingroup

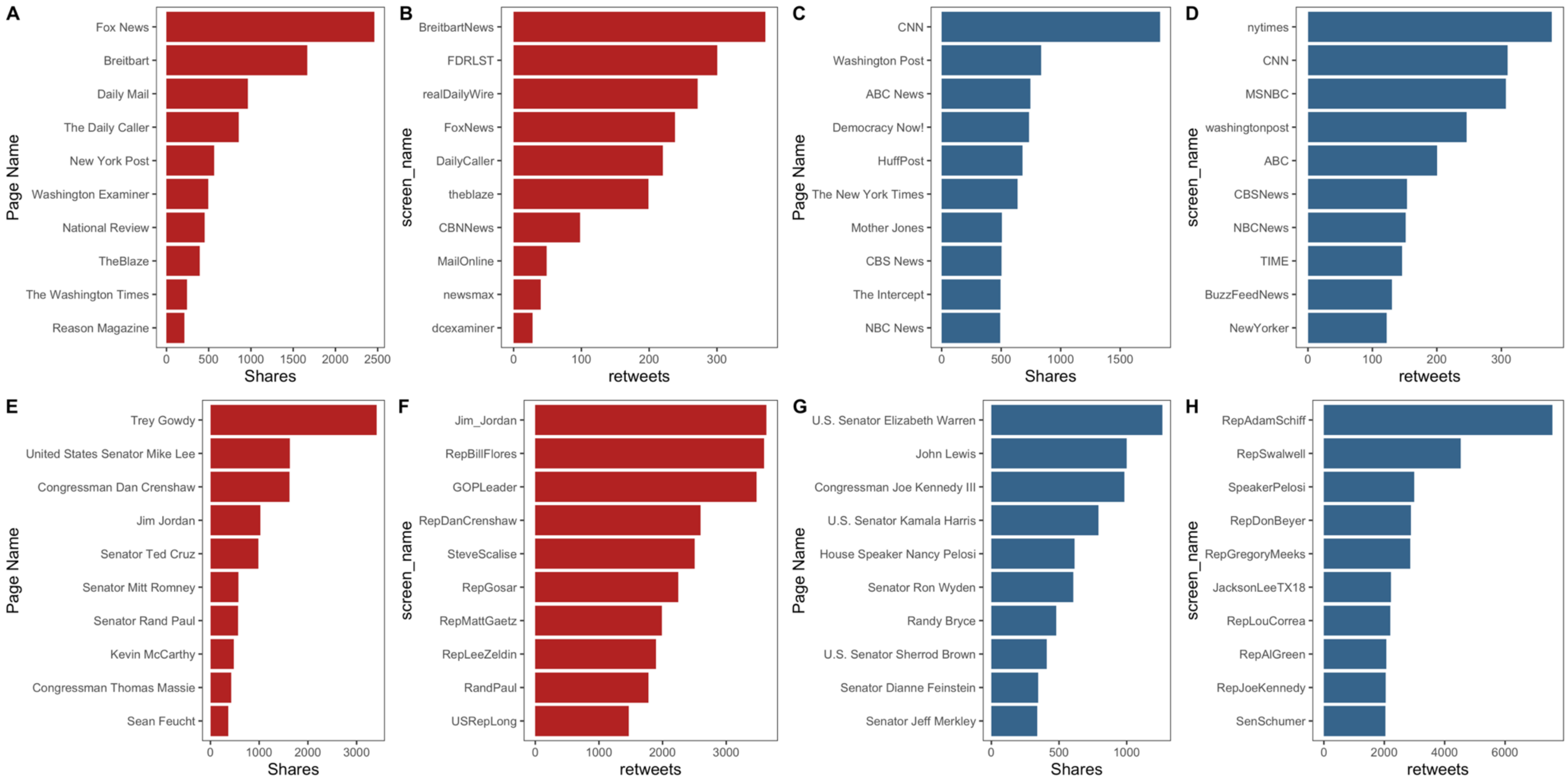

Figure S1. Pages Associated with the Most Engagement on Facebook and Twitter. Panels represent (A) conservative media Facebook, (B) conservative media Twitter, (C) liberal media Facebook, (D), liberal media Twitter, (E) conservative congress Facebook, (F) conservative congress Twitter, (G) liberal congress Facebook, and (H) liberal congress Twitter.

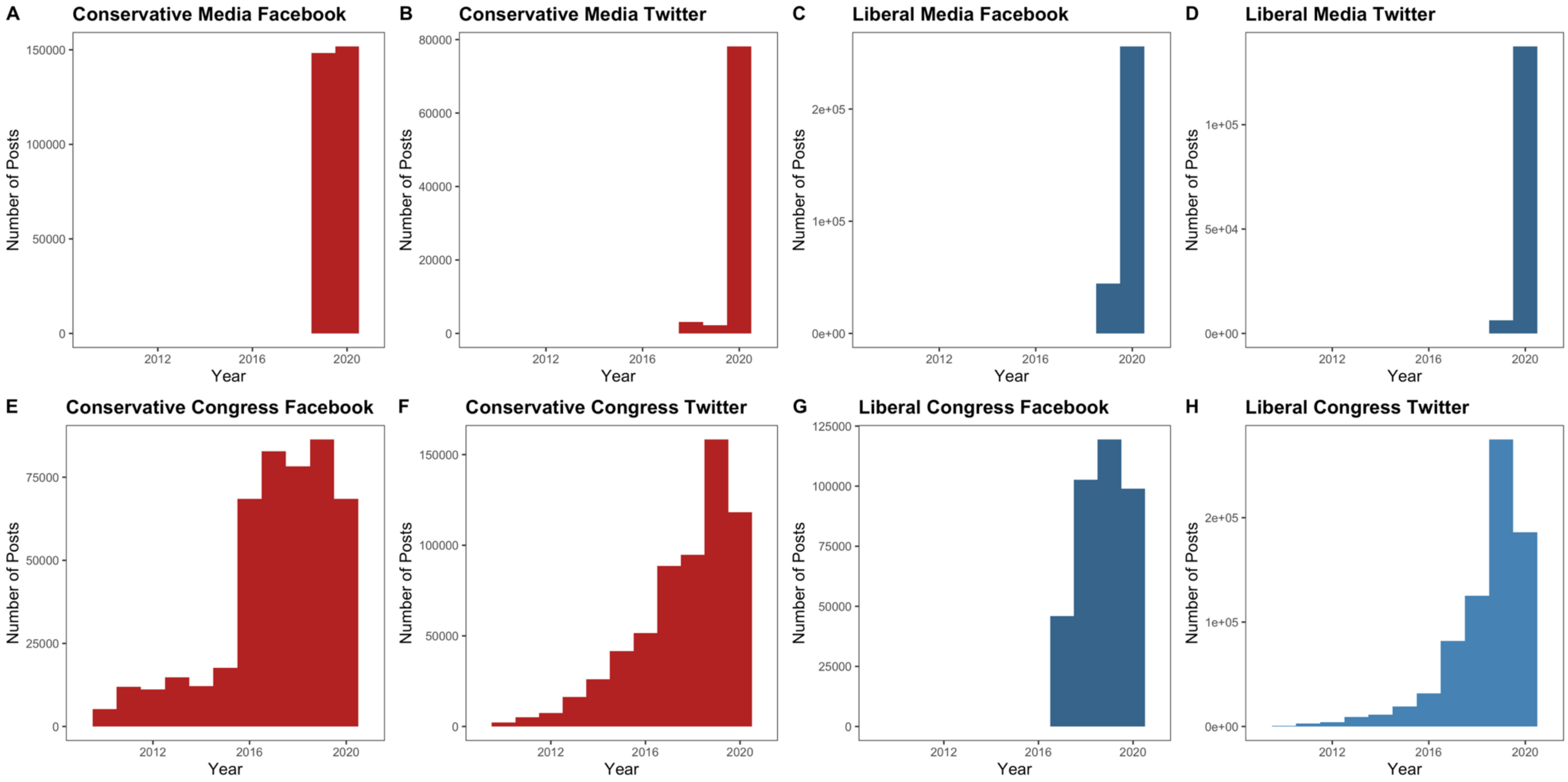

Figure S2. Histograms of the time the tweets and Facebook posts were created. The media tweets were retrieved on May 4 and July 11, 2020; the congress tweets were retrieved on July 2, 2020; the media Facebook posts were retrieved on August 14, 2020, and the congress media posts were retrieved on August 18, 2020.

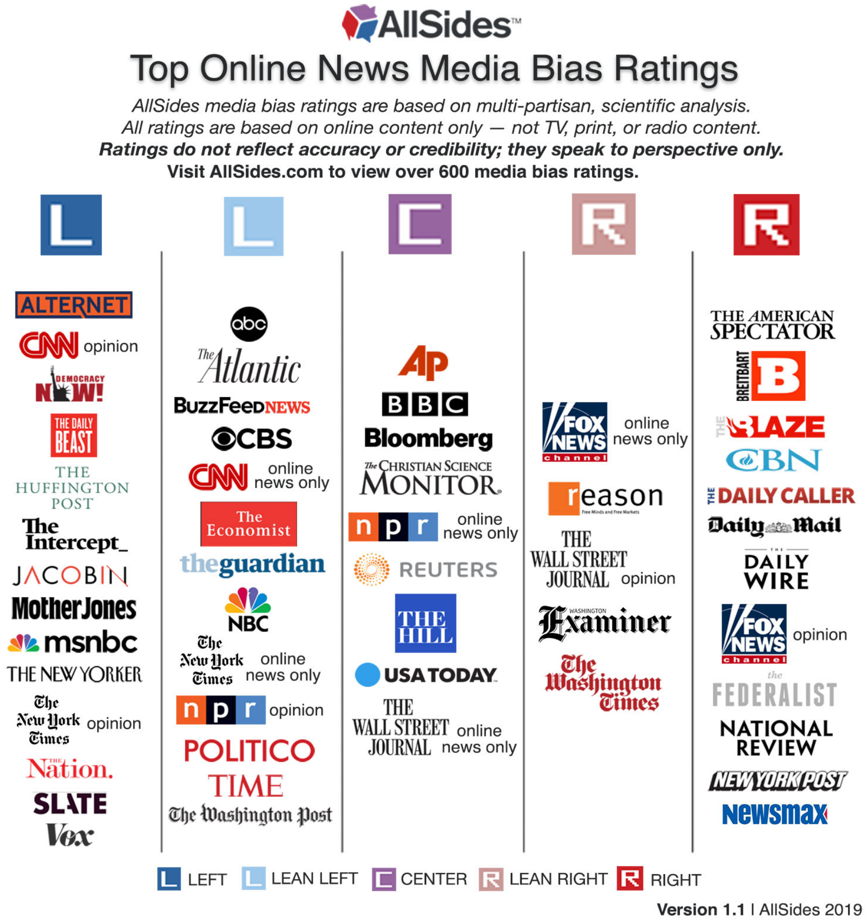

Figure S3: AllSides Media Bias Chart. The above 2019 AllSides Media Bias Chart (retrieved from AllSides.com) was used retrieve Twitter handles and Facebook accounts. The left and right media accounts (but not the centrist ones) were used.
